# The evolution and genetic diversity of avian influenza A(H9N2) viruses in Cambodia, 2015 – 2016

**DOI:** 10.1371/journal.pone.0225428

**Published:** 2019-12-09

**Authors:** Annika Suttie, Songha Tok, Sokhoun Yann, Ponnarath Keo, Srey Viseth Horm, Merryn Roe, Matthew Kaye, San Sorn, Davun Holl, Sothyra Tum, Ian G. Barr, Aeron C. Hurt, Andrew R. Greenhill, Erik A. Karlsson, Dhanasekaran Vijaykrishna, Yi-Mo Deng, Philippe Dussart, Paul F. Horwood

**Affiliations:** 1 Virology Unit, Institut Pasteur du Cambodge, Institut Pasteur International Network, Phnom Penh, Cambodia; 2 School of Health and Life Sciences, Federation University, Churchill, Australia; 3 WHO Collaborating Centre for Reference and Research on Influenza, Victorian Infectious Diseases Reference Laboratory, Peter Doherty Institute for Infection and Immunity, Melbourne, Victoria, Australia; 4 National Animal Health and Production Research Institute, General Directorate of Animal Health and Production, Cambodian Ministry of Agriculture, Forestry and Fisheries, Phnom Penh, Cambodia; 5 Department of Microbiology and Immunology, The University of Melbourne, Peter Doherty Institute for Infection and Immunity, Melbourne, Victoria, Australia; 6 Department of Microbiology, Biomedicine Discovery Institute, Monash University, Melbourne, Victoria Australia; 7 College of Public Health, Medical and Veterinary Sciences, James Cook University, Townsville, Australia; St. Jude Children's Research Hospital, UNITED STATES

## Abstract

Low pathogenic A(H9N2) subtype avian influenza viruses (AIVs) were originally detected in Cambodian poultry in 2013, and now circulate endemically. We sequenced and characterised 64 A(H9N2) AIVs detected in Cambodian poultry (chickens and ducks) from January 2015 to May 2016. All A(H9) viruses collected in 2015 and 2016 belonged to a new BJ/94-like h9-4.2.5 sub-lineage that emerged in the region during or after 2013, and was distinct to previously detected Cambodian viruses. Overall, there was a reduction of genetic diversity of H9N2 since 2013, however two genotypes were detected in circulation, P and V, with extensive reassortment between the viruses. Phylogenetic analysis showed a close relationship between A(H9N2) AIVs detected in Cambodian and Vietnamese poultry, highlighting cross-border trade/movement of live, domestic poultry between the countries. Wild birds may also play a role in A(H9N2) transmission in the region. Some genes of the Cambodian isolates frequently clustered with zoonotic A(H7N9), A(H9N2) and A(H10N8) viruses, suggesting a common ecology. Molecular analysis showed 100% of viruses contained the hemagglutinin (HA) Q226L substitution, which favours mammalian receptor type binding. All viruses were susceptible to the neuraminidase inhibitor antivirals; however, 41% contained the matrix (M2) S31N substitution associated with resistance to adamantanes. Overall, Cambodian A(H9N2) viruses possessed factors known to increase zoonotic potential, and therefore their evolution should be continually monitored.

## Introduction

Subtype A(H9) avian influenza viruses (AIVs) circulate globally in wild avian species and are endemic in domestic poultry in many Asian, Middle Eastern and African countries [1,2]. Despite its status as low pathogenic avian influenza (LPAI), subtype A(H9) presents a concern for both the agricultural and health sectors. While infected flocks only experience mild respiratory disease with mortality rates generally below 20% [3,4], A(H9) infections decrease body weight of broilers and the egg production and quality in layers and breeders [5,6]. Additionally, infected poultry are rendered more susceptible to secondary infections [7–9], potentially increasing flock mortality levels up to 65% [10,11].

A(H9) AIVs in poultry pose a zoonotic risk to humans [12]. Since the first official report of a human A(H9N2) case in 1998, 59 additional cases have been reported from Bangladesh, China, Egypt, Pakistan and Oman [13]. Human A(H9) infections are generally asymptomatic or manifest as mild respiratory disease. Only one A(H9N2)-associated death has been reported, likely due to comorbidities [14]. All A(H9N2) viruses detected in humans are genetically similar to poultry viruses from the same period [13,15–17]. A(H9) AIVs also commonly donate internal genomic segments to non-A(H9) viruses through reassortment, creating unique AIV genotypes that may present a threat for agricultural sectors and human health [18–21]. This phenotype has been observed for A(H5N1), A(H5N2), A(H5N6), A(H7N7), A(H7N9) and A(H10N8) subtype AIVs [18,22,23]. Of particular interest, A(H5N6), A(H7N9) and A(H10N8) AIVs all acquired internal segments from genotype 57 (G57) A(H9) viruses before emerging as zoonotic viruses [18–22]. G57 is generally equivalent to genotype S [4], the most prevalent A(H9) genotype detected in China since 2013 [1,18].

Cambodian AIV surveillance started in response to the detection of highly pathogenic avian influenza (HPAI) A(H5N1) in Cambodian poultry in 2004. These viruses now circulate endemically in domestic poultry species [24,25]. In 2013, surveillance efforts expanded to encompass A(H7) and A(H9) viruses [26–28]. It is now evident that A(H9) LPAI viruses circulate endemically in Cambodian poultry [24,28,29] similar to Bangladesh, China and Vietnam [30–32] and live bird market (LBM) workers are exposed to these viruses [24]. Therefore, A(H9) AIVs pose a significant risk to Cambodian agricultural and public health sectors. In order to further understand the diversity, origins and molecular risk traits of A(H9) AIVs circulating in Cambodian poultry, we analyzed full genomic sequences of A(H9N2) viruses identified in poultry from LBMs between 2015 and 2016.

## Materials and methods

### Sample collection

Active AIV surveillance in Cambodia was performed by Institut Pasteur du Cambodge (IPC) in collaboration with the National Animal Health and Production Research Institute (NAHPRI) under the direction of the General Directorate for Animal Health and Production, Cambodian Ministry of Agriculture, Forestry and Fisheries (MAFF). Throughout 2015 and 2016 surveillance for AIVs in poultry was conducted at two prominent LBMs in the Cambodian poultry network: i) Orussey market located in Phnom Penh, the capital city of Cambodia; and, ii) Takeo market, a provincial market located in Takeo province (S1 Fig) [33]. In 2015, samples were collected weekly from February to December [28]. In 2016, collections targeted periods known for having high levels of AIV circulation: Lunar New Year (February), Khmer New Year (April) and Pchum Ben (October). Oropharyngeal and cloacal samples from individual birds were pooled and screened for A(H9) AIVs with qRT-PCR assays sourced from the International Reagent Resource (https://www.internationalreagentresource.org/Home.aspx) as described previously [27,28].

### Viral isolation

A subset of A(H9) AIV positive samples, with matrix gene qRT-PCR Ct values <30, were isolated in embryonated chicken eggs (ECEs) [34]. Briefly, original pooled samples were diluted 1:1 with a penicillin-streptomycin PBS solution, filtered (0.22 μM) and then inoculated into the allantoic cavity of 10–12 day old ECEs. Allantoic fluid was collected 48–72 hours post inoculation. The presence of AIV was verified by hemagglutination assay with 0.5% chicken red blood cells followed by qRT-PCR for the AIV MP gene. Negative samples were passaged a minimum of three times in ECEs, after which viral isolation was deemed unsuccessful.

### Sequencing of viral isolates

A(H9N2) AIV positive isolates were sent to the WHO Collaborating Centre for Reference and Research on Influenza, Melbourne, Australia for whole genome sequencing (WGS). Prior to sequencing, RNA was extracted from samples with the NucleoMag® VET kit (Macherey-Nagel) and all genomic segments were amplified in a single reverse transcription PCR (RT-PCR) reaction [35]. The standard manufacturer’s protocol was used to obtain whole genome sequences using the Ion Torrent PGM. Briefly, the concentration of RT-PCR products was normalised and fragmented to produce a 200 base-read library using the Ion Xpress^™^ Plus Fragment Library Kit (Life Technologies). Samples were barcoded with unique Ion Xpress barcode adapters (Life Technologies), pooled and purified using AMPure XP reagent (Agencourt). The final libraries were quantified using the Ion Library Quantitation Kit (Life Technologies) and 20 pM was used to perform an emulsion PCR to enrich Ion Sphere Particles^™^ (ISPs) on the Ion OneTouch 2 instrument (Life Technologies). The ISPs were then loaded onto a 318^™^ Chip v2 (Life Technologies) and run on the Ion Torrent PGM.

Quality control of the NGS reads was assessed using CLC Genomic Workbench v10 (QIAGEN). Low quality reads less than 50 nucleotides in length were removed and a minimum base call quality Phred score of 20 was set. Remaining sequences were aligned to a reference genome (GISAID accession numbers: EPI542434, EPI542395, EPI542405- EPI542410). Genes containing gaps or areas of low sequence coverage were completed using Sanger sequencing. Briefly, segment specific primers were used to amplify appropriate regions using conventional PCR [36]. Sequencing was performed using Big Dye Terminator Reaction Mix (Applied Biosystems) on an ABI 3500xL Genetic Analyzer. The NGS and Sanger sequencing data was assembled to produce consensus sequences using Geneious® 9.1.8 (Biomatters Ltd). Sequence accession numbers for viruses generated during this study are available in S1 Table.

### Phylogenetic analysis

Sequences included in the phylogenetic analysis were obtained from GISAID [37], GenBank [38] or the Influenza Research Database [39] and aligned along with Cambodian A(H9N2) viral sequences using MAFFT v7.308 [40]. IQ-Tree was used to produce Maximum Likelihood (ML) phylogenetic trees using the best-fit nucleotide substitution model defined by the Akaike Information Criteria [41]. The General Time Reversible nucleotide substitution model with invariant sites (I) and gamma rate of heterogeneity (GTR + I + Γ) was selected as the best-fit for all datasets, except for MP and NS that used the transversion model (TVM) + I + Γ. Topological support was estimated by 1,000 ultrafast bootstrap replicates [42,43].

Time to the most recent common ancestor (TMRCA) for each node of the HA and NA phylogenies was estimated using a Bayesian Markov Chain Monte Carlo (MCMC) approach with BEAST v1.8.4 run on the CIPRES Science Gateway web portal [44]. For each gene the GTR + Γ and SRD06 nucleotide substitution model was used [45] with a relaxed uncorrelated log-normal molecular clock [46]. Population dynamics was investigated by using a coalescent Gaussian Markov random field Bayesian skyride tree prior with time-aware smoothing [47]. Maximum clade credibility (MCC) tree for each gene was the result of two independent analysis runs for 100 million generations, sampled to produce 10,000 states with 10% removed as burn-in and combined using TreeAnnotator v1.8.4. The ML and MCC trees were formatted using FigTree v1.4.3 [48].

Reassortment was visualised by producing a phylogenetic congruency map, whereby segments of the individual viruses were linked across the ML phylogenies. The ML trees were created using IQ-tree as described above with a subset of viruses that had sequencing data available for all eight genomic segments (S2 Table). Incongruence is demonstrated by deviations in the topology and can indicate that reassortment has occurred.

### Viral genotyping

For the purpose of this study, the genotyping system used follows conventions initially described by Li et al., 2005 [3]. Using this system A(H9) AIVs are separated into genotypes designated A through to W based on the phylogenetic relationship of genes to eight reference viruses: A/turkey/Wisconsin/1/1966 (WI/1/66), A/duck/Hong Kong/d73/1976 (HK/d73/76), A/chicken/Beijing/1/1994 (BJ/1/94), A/chicken/Korea/38349-p96323/1996 (KR/96323/96), A/chicken/Hong Kong/G9/1997 (G9/97), A/duck/Hong Kong/Y439/1997 (Y439/97), A/quail/Hong Kong/G1/1997 (G1/97) and A/chicken/Shanghai/F/1998 (F/98) (for review see reference [49]).

### Molecular analysis

The H3 HA and N2 NA numbering systems were used throughout the text, unless otherwise stated. Molecular markers associated with changes in viral fitness or resistance to antivirals were investigated using the molecular inventory matrix produced by Suttie et al., 2019 [50]. N-linked glycosylation sites for HA and NA were predicted using the NetNGlyc1.0 server with default settings [51].

### Selection pressure analysis

Selection pressures acting on each gene of the Cambodian A(H9N2) AIVs was analysed by calculating the ratio of non-synonymous to synonymous mutations (dN/dS ration, ω) using the HyPhy software package [52] accessed via the datamonkey webserver [53]. Selection pressure is interpreted based on the value of ω: ω < 1 indicates negative selection, ω = 1 neutrality and ω > 1 positive selection. Programs used to infer selection pressure included: fixed-effects likelihood (FEL), fast unconstrained Bayesian approximation (FUBAR), mixed effects model of evolution (MEME) and single-likelihood ancestor (SLAC) [54–56]. Statistically significant sites detected using two or more programs with a p-value < 0.1 for FEL, MEME or SLAC, or a posterior probability ≥0.90 for FUBAR were considered valid.

### Neuraminidase inhibition assay

The susceptibility of 40 Cambodian A(H9N2) viruses to four NA inhibitors (NAIs; oseltamivir, zanamivir, laninamivir and peramivir) was evaluated using a standard fluorescence based assay that measures NA enzymatic activity to determine the 50% inhibitory concentration (IC_50_) of viruses. This protocol has been described in detail by Leang et al., 2017 [57]. The IC_50_ values were calculated using the JASPR v1.2 software (CDC, Atlanta, USA). NAI susceptibility is described based on the fold change in IC_50_ values of the test viruses compared to control viruses; viruses with normal inhibition had <10-fold increase in their IC_50_, reduced inhibition had between 10 to 100-fold increase in IC_50_ or highly reduced inhibition >100-fold increase in IC_50_.

### Ethical approval

Animal sampling was conducted by the National Animal Health and Production Research Institute under the direction of the General Directorate for Animal Health and Production, Cambodian Ministry of Agriculture, Forestry and Fisheries as part of routine disease surveillance activities; thus, poultry sampling was not considered as experimental animal research. The analysis of poultry samples for avian influenza testing was conducted by the Virology Unit at Institut Pasteur du Cambodge as part of routine activities as a World Health Organization H5 Reference Laboratory.

## Results

### Phylogenetic diversity of the Cambodian A(H9N2) viruses

**Fig 1:**
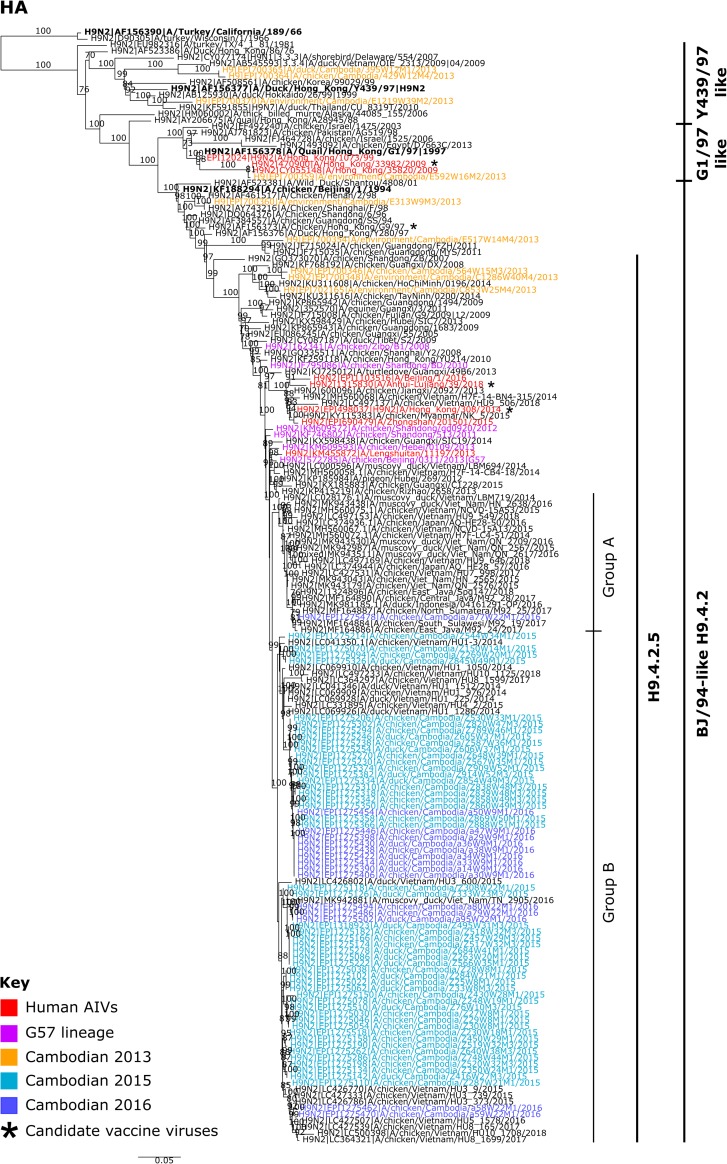
Maximum likelihood phylogenetic tree of the A(H9) HA gene. The tree was produced using IQ-Tree with the GTR+ I + Γ model. Phylogenetic support was estimated using 1,000 ultrafast bootstrap replicates. Bootstrap values greater than 70% are displayed on branches. Cambodian A(H9) viruses are coloured according to the year they were detected: 2013 is shown in orange, 2015 in light blue and 2016 in dark blue. AIVs detected in humans are indicated in red and G57 viruses, as determined by Pu et al., 2015 [18], are shown in pink. Candidate vaccine viruses are indicated by an asterisks (*). Reference viruses are indicated in bold and viral lineages are indicated on the right of the phylogeny. Scale bars indicate the number of nucleotide substitutions per site.

A total of 64 A(H9N2) viruses (49 in 2015; and 15 in 2016) were isolated in ECEs, with 43 from chickens and 21 from ducks (S1 Table). Further analyses were performed on full genomes of 63 isolates and the partial genome of one isolate (A/duck/Cambodia/Z495W31M3/2015). Phylogenetic analysis of the Cambodian A(H9N2) viruses showed that the diversity of A(H9) AIVs decreased from 2013, when all three main H9-HA lineages (Y439/97, G1/97 and BJ/94) were identified (Fig 1). Only the BJ/94 lineage was detected in 2015 and 2016. Despite arising from the same H9-4.2.5 clade [58], the 2015/16 HA genes (nucleotide identities 95.5–100%) did not cluster with 2013 viruses indicating that HA replacements occurred between the surveillance periods.

A single virus, designated A/chicken/Cambodia/a77W22M1/2016 (Group A), clustered independently with a lineage predominantly containing A(H9N2) viruses collected from: Vietnamese poultry from 2014 to 2018, a single virus detected in a Japanese chicken from 2016 and Indonesian poultry from 2016 to 2018. The remaining viruses (n = 63, Group B) formed a monophyletic lineage with Vietnamese viruses identified from 2014 to 2018 with multiple, independent, region specific clusters indicating frequent cross-border movement of A(H9N2) (Fig 1). All HA genes diverged from a common ancestor around September 2011 with a 95% Highest Posterior Density (HPD) interval from January 2011 to June 2012 (S2A Fig) indicating dispersion from an original Asian source. However, predominant Group B viruses diverged more recently during May 2013 (95% HPD, September 2012—December 2013).

**Fig 2:**
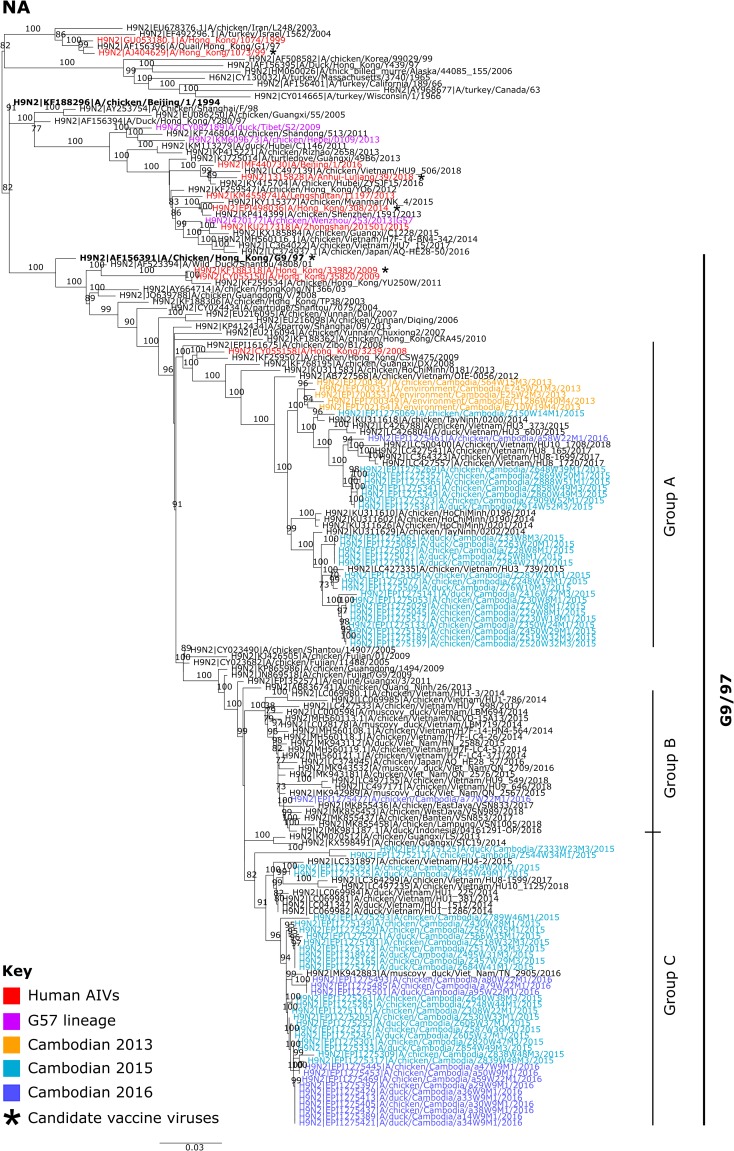
Maximum likelihood phylogenetic tree of the N2 NA gene. The tree was produced using IQ-Tree with the GTR+ I + Γ model. Phylogenetic support was estimated using 1,000 ultrafast bootstrap replicates. Bootstrap values greater than 70% are displayed on branches. Cambodian A(H9) viruses are coloured according to the year they were detected: 2013 is shown in orange, 2015 in light blue and 2016 in dark blue. AIVs detected in humans are indicated in red and G57-like viruses, as determined by Pu et al., 2015 [18], are shown in pink. Candidate vaccine viruses are indicated by an asterisks (*) to the right of the taxa name. Reference viruses are indicated in bold and viral lineages are indicated on the right of the phylogeny. Scale bars indicate the numbers or nucleotide substitutions per site.

The NA genes of Cambodian A(H9N2) viruses showed a greater genetic diversity (mean TMRCA April 2005, 95% HPD August 2004–December 2005) (S2B Fig) with three main clusters (Fig 2), all in the G9/97 NA reference lineage. Group A contained all Cambodian A(H9N2) clade h9-4.2.5 viruses from 2013 (n = 5), and a subset of 2015 (n = 25) and 2016 (n = 1) viruses. Persistence of the N2 lineage containing viruses from 2013, but not associated HA lineages, suggests reassortment between the previously circulating and newly introduced h9-4.2.5 viruses. N2 Group A stemmed from A(H9N2) viruses identified in poultry from Hong Kong SAR (China) and mainland China in 2008/09, as well as a single A(H9N2) virus identified in a human from Hong Kong SAR (China) in 2008. Within this cluster, Cambodian A(H9N2) AIVs detected between 2013 to 2016 formed a monophyletic lineage with viruses identified in Vietnamese poultry from 2012 to 2018. N2 Group B contained a single Cambodian isolate, A/chicken/Cambodia/a77W22M1/2016, which clustered similarly to HA Group A with viruses from Indonesia, Japan and Vietnam. Finally, N2 Group C contained some Cambodian A(H9N2) viruses from 2015 (n = 24) and the majority of 2016 isolates (n = 13). Similarly to N2 Group A, this clade stemmed from A(H9N2) viruses identified in China and clustered closely with viruses identified in Vietnamese poultry from 2014 to 2018 (nucleotide identity 89.3–100%).

Bayesian estimates of the TMRCA for the Cambodian A(H9N2) H9 HA and N2 genes indicate that the H9 HA genes share a more recent common ancestor than the N2 genes, demonstrating there may have been a greater turnover of H9 HA lineages compared to N2. Additionally, Bayesian skyride analysis of genetic diversity shows greater fluctuations in H9 HA diversity compared to N2 (S3A–S3B Fig). Overall, the Bayesian MCC and ML phylogenies reveal there has been reassortment between persistent NA lineages with newly introduced HA segments into Cambodia.

**Fig 3:**
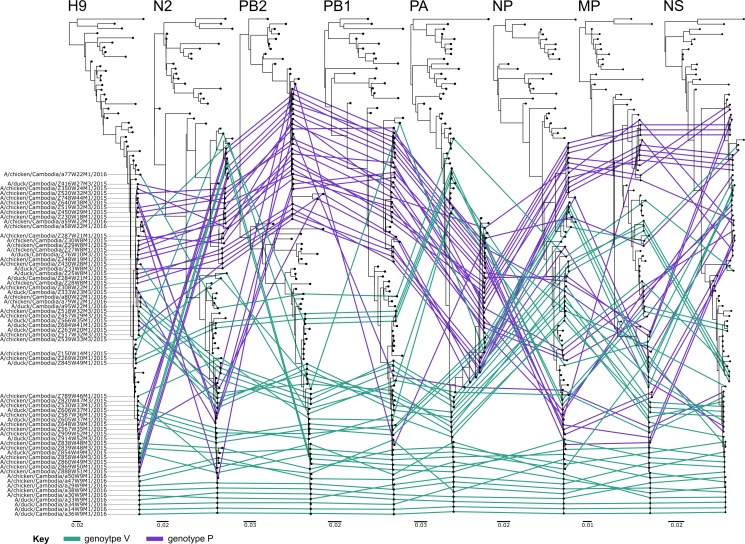
Phylogenetic congruency of Cambodian A(H9N2) viruses. ML trees were produced for all genomic segments using all Cambodian A(H9N2) viruses detected from 2015 to 2016 and key reference viruses. The phylogenies of the Cambodian viruses have been linked across all eight segments and colour coded according to viral genotype, genotype P viruses are shown in purple and genotype V in green. Viral designations are listed on the left hand side of the HA phylogeny. Incongruence is demonstrated by deviations in topology across the segments and can indicate viral reassortment has occurred. Scale bars are shown below each phylogeny and indicate the number of nucleotide substitutions per site.

Similar to HA and NA, internal genes of the Cambodian A(H9N2) viruses also split into either two or three main groups (S4A–S4F Fig). However, group composition is not consistent across the different segments, further indicating extensive reassortment between co-circulating lineages. This is also evident by the phylogenetic incongruence shown between segments of many viruses (Fig 3; nucleotide sequence identities: PB2: 83.6–100%, PB1: 92.9–100%, PA: 92.7–100%, NP: 90.8–100%, MP: 93.5–100% and NS: 92.7–100%). For instance, the virus A/chicken/Cambodia/a58W22M1/2016 displays incongruence (Fig 3). This is a genotype V virus with congruence links in Fig 3 shown in green, however its HA and PB1 genes clearly deviate to cluster with genotype P viruses shown in purple.

All Cambodian A(H9N2) gene phylogenies, excluding NA, cluster with lineages containing zoonotic A(H7N9), A(H9N2) and A(H10N8) AIVs (Figs 1, 2 and S4A–S4F). Typically, Cambodian A(H9N2) AIVs did not cluster closely with the genotype S zoonotic viruses. However, a single isolate, A/chicken/Cambodia/a77W22M1/2016, had PB2, PA, MP and NS genomic segments similar to AIVs that have infected humans (nucleotide sequence identities >98%; S4A, S4C, S4E and S4F Fig). This virus clustered separately from other Cambodian A(H9N2) isolates, representing a possible separate introduction of A(H9N2) into Cambodia. This cluster contained AIVs from China and Japan, as well as waterfowl hosts in Vietnam, and is most closely related to A(H9N2) AIVs detected in Indonesia, with all genomic segments having a nucleotide sequence identity >99%.

### Genotypes of Cambodian A(H9N2) viruses

**Fig 4:**
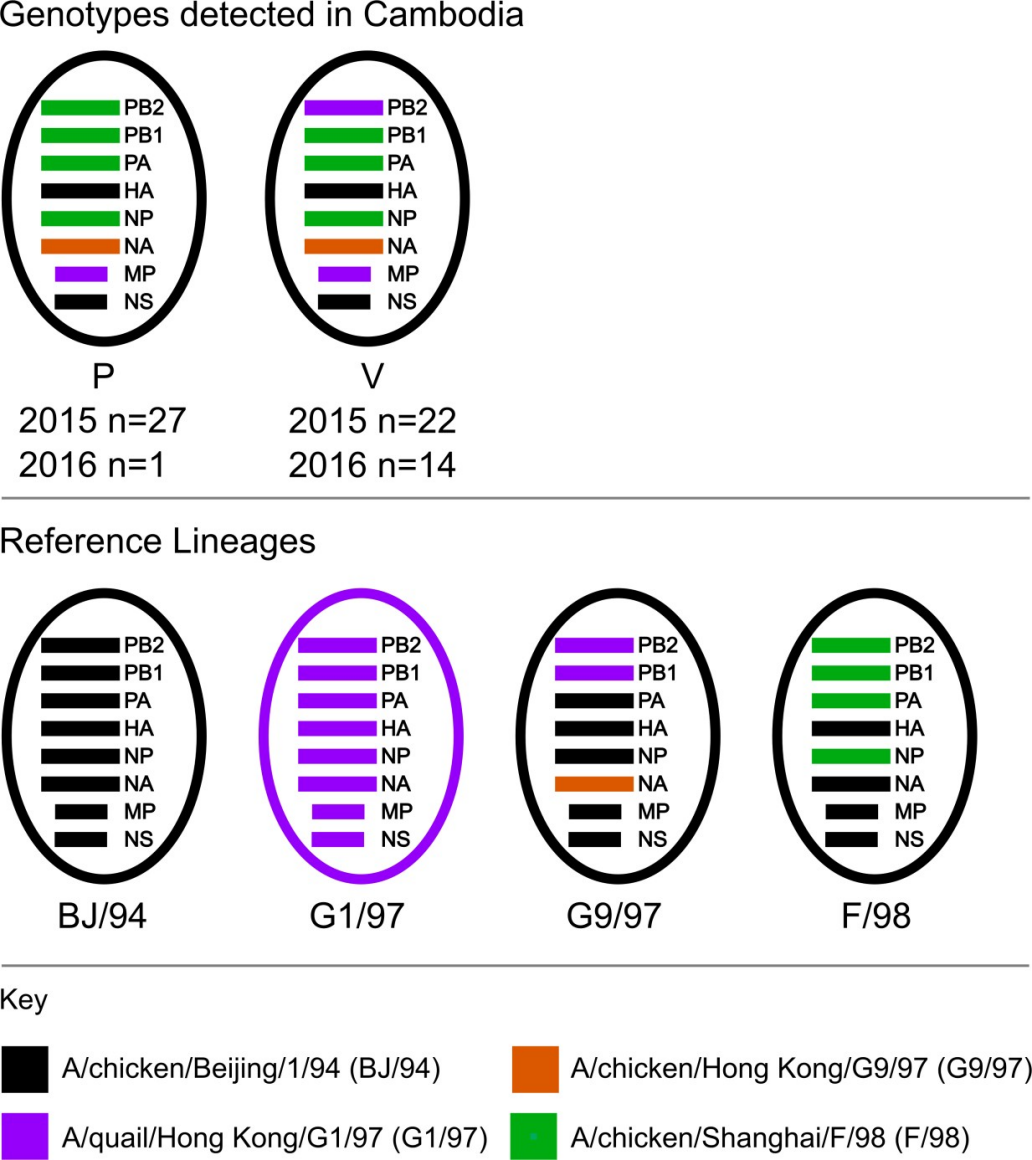
Genotypes of A(H9N2) Cambodian viruses detected from 2015 to 2016. The eight genomic segments are colour coded based on the putative ancestral reference AIVs. The genomic constellations of relevant reference lineage viruses (BJ/94, G1/97, G9/97 and F/98) are shown in the bottom panel. Segments from BJ/94 viruses are shown in black, G1/97 in purple, G9/97 in orange and F/98 in green.

The 64 Cambodian A(H9N2) viruses isolated during this study belonged to two genotypes, P (n = 28) and V (n = 36). Initially described in Chinese poultry, each genotype stems from multiple reassortment events of A(H9) viruses from the G1/97, G9/97, BJ/94 and F/98 viral lineages (Fig 4) [1,59]. Genotype P viruses, represented by A/chicken/Zibo/B1/2008, were first identified in 2008. They had an F/98 backbone, a G9/97 N2 segment and a G1/97 MP segment [4,59]. Genotype V viruses, represented by A/chicken/Guangxi/SIC19/2014, were first identified in 2014 and are the result of reassortment of genotype S viruses with the G9/97 lineage [1,4]. These viruses have an F/98 backbone with PB2 and MP genes from the G1/97 lineage and a G9/97 NA segment. In Cambodia, genotype P and V were both detected in 2015 and 2016 (Fig 4), and have previously been detected in Vietnam [30]. In Cambodia, the detection rates of each genotype were similar in 2015, P (n = 27) and V (n = 22). However, in 2016, genotype V predominated (n = 14) versus P (n = 1).

### Cambodian A(H9N2) viruses have molecular markers associated with viral adaptation to mammals

Screens for mutations known to affect AIV pathogenicity (S3A–S3H Table), are summarised in Table 1 [50]. All Cambodian viruses had HA cleavage site motifs common for A(H9) LPAI viruses: PSKSSR/GLF (n = 55) and PSRSSR/GLF (n = 9) [60]. The HA genes also contained a number of amino acid substitutions associated with an increase in AIV binding to human-type α2,6 receptors (Table 1 and 32d), including Q226L in 100% of Cambodian A(H9N2) isolates.

**Table 1:** Summary of amino acid substitutions in Cambodian A(H9N2) viruses associated with an increase in viral fitness.

Protein	Phenotype	Mutation/Motif	Residues for Cambodian Isolates (%)	References
*PB2*	Increased polymerase activity and replication in mammalian cell lines, increase virulence in mice	A588V	V (2)	[61]
		V598T	T (44)	[62]
		Q591K	K (5)	[63]
		E627K	E (100)	[64,65]
		D701N	D (100)	[65]
*PB1*	Increased polymerase activity and virulence in mice	D622G	G (100)	[66]
*PB1-F2*	Truncations to the 90 aa protein increase AIV pathogenicity in chickens	90aa	90aa (95)	[67]
			76aa (2)	
			8aa (3)	
*PA*	Increased polymerase activity and replication in mammalian cell lines, increased virulence in mice	V63I	I (100)	[68]
		K356R	R (2)	[69]
*PA-X*	Full length PA-X increases A(H9) virulence in mice	252aa	252aa (100)	[70]
*HA*	Multibasic cleavage site can increase viral pathogenicity	Mono-basic	PSKSSR/GLF (86)	[71,72]
			PSRSSR/GLF (14)	
	Increased specificity for α2,6 human-type receptors	D101N	N (98)	[73]
		I155T*	T (100)	[74]
		S158N	N (36)	[75]
		E/T190V	V (9)	[76]
		N248D	D (3)	
		R496K*	K (100)	[77]
	Increased specificity for α2,6 human-type receptors, enhanced replication in mammalian cells and ferrets, enhanced contact transmission in ferrets	Q226L	L (100)	[78,79]
*NP*	Increased virulence in chickens	M105V	V (91)	[80]
*M1*	Increased virulence in mice, chickens and ducks	I43M	M (100)	[81]
	Increased virulence in mice	T139A	A (100)	[82,83]
*M2*	Increased resistance to amantadine and rimantadine	S31N	N (41)	[84]
*NS1*	Decreased antiviral response and increased virulence in mice	P42S	S (100)	[85]
	Decreased interferon response and increased virulence in chickens	V149A	A (100)	[86]

* Amino acid (aa) mutations where the phenotype is produced only when in combination with other mutations

Cambodian A(H9N2) viruses contained numerous substitutions associated with increased polymerase activity of AIVs in mammalian cell lines (Tables 1, S3A–S3C and S3E). However, well known substitutions in PB2, namely E627K and D701N, were not detected. The majority (n = 61) of viruses had full length, 90 aa PB1-F2. A single virus, A/chicken/Cambodia/a77W22M1/2016, had a minor PB1-F2 truncation and was 76 aa long. Additionally, two viruses had major truncations with premature stop codons at position 9. All Cambodian viruses had full length PA-X proteins at 252 aa in length.

Only a small number of mutations in MP and NS1 were detected that increase AIV virulence in mammalian or avian models (Tables 1 and S3G–S3H). Cambodian A(H9N2) viruses contained either truncated (217 aa, n = 36) or N-terminal elongated (237 aa, n = 28) NS1 protein. NS1 elongation likely arose in a specific cluster within the Cambodian A(H9N2) population (S4F Fig). No molecular markers of concern were identified in NS2 (Tables 1 and S3H). All MP genes contained I43M and T215A, associated with increase virulence in mice (Tables 1 and S3G).

### Similarity of Cambodian A(H9N2) viruses to available vaccine strains

Genetic analysis of known A(H9) HA antigenic sites [87] showed high similarity between Cambodian A(H9N2) viruses. Of the 33 sites investigated, Cambodian isolates had a maximum of 4 amino acid differences (S4 Table). Compared to available poultry vaccine viruses (A/chicken/Shandong/6/96 (6/96), A/chicken/Guangdong/SS/94 (SS/94), F/98), Cambodian A(H9N2) AIV HA was most similar to SS/94 (nucleotide sequence similarity: 90.5% to 91.8%, 6 to 9 antigenic aa differences; S4 Table). Compared to the human candidate vaccine viruses (CVVs) (G9/97, A/Hong Kong/1073/99, A/Hong Kong/33982/2009, A/Hong Kong/308/2014, A/Anhui-Lujiang/39/2018), Cambodian viruses were most similar to A/Hong Kong/308/2014 at a nucleotide level (sequence similarity: 94.1% to 95.1%). Although, they were more similar to G9/97 at H9 HA antigenic sites with 4 to 7 aa differences, compared to A/Hong Kong/308/2014 that had 8 to 11 aa differences.

### Post translational modifications

In the Cambodian A(H9N2) viruses, seven conserved HA glycosylation sites were predicted, including: ^11^NSTE, ^280^NTTL, ^287^NVSK/R, ^295^NCSK and ^474^NGTY in the HA stalk domain, as well as ^123^NVSY and ^200^NRTF in the HA globular domain (S5 Table). More variation was observed in glycosylation sites predicted for N2. Three glycosylation sites (positions: 44, 61 and 146) were conserved in the N2 gene of all Cambodian viruses. Variable sites were identified at positions: 48, 69, 70, 86, 234 and 306. The majority of isolates contained 6 predicted glycosylation sites in NA (n = 40), though isolates with 7 (n = 15), 5 (n = 8) and 4 (n = 1) sites were also identified.

### Selection pressure

Selection pressure analysis showed that the majority of sites in each gene of Cambodian A(H9N2) viruses were under neutral or negative selection. A small number of codons in HA (78, 158), NA (9, 12, 416), PB2 (389, 758), M2 (21, 97) and NS2 (119) were under positive selection (S6 Table). Substitutions at the majority of these sites are not associated with increased AIV fitness. However, substitution of serine for asparagine (S158N) at HA codon 158 has been shown to increase AIV binding to human-type α2,6 receptors in A(H5) viruses [75,87]. Additionally, PB2 K389R increases polymerase activity and replication of A(H7) AIVs in mammalian cell lines [62]. These phenotypes have not been examined in A(H9) AIVs and require further investigation.

### The susceptibility of Cambodian A(H9N2) viruses to antiviral drugs

Molecular analysis of the M2 protein from Cambodian A(H9N2) viruses showed 41% (n = 26) had the S31N marker associated with resistance to adamantanes [84]. Mapping this mutation to the MP phylogeny indicates that the S31N has arisen in the population on multiple independent occasions (S4E Fig). None of the Cambodian viruses contained PA I38T/F/M mutations associated with resistance to the recently licenced cap dependent endonuclease antiviral, baloxavir marboxil [88,89]. None of the Cambodian A(H9N2) AIVs harboured known N2 mutations associated with resistance to NAIs. Susceptibility to NAIs was confirmed in 40 Cambodian A(H9N2) viruses with IC_50_ values <10 against oseltamivir, zanamivir, laninamivir and peramivir (S7 Table).

## Discussion

Since integration of detection protocols in 2013, it is clear that subtype A(H9) AIVs now circulate endemically in Cambodian domestic poultry and they are currently the most prevalent subtype detected [29]. Between 2013 and 2016, the genomic diversity of Cambodian A(H9) HA genes decreased, with 2015–2016 viruses belonging to a single clade, BJ/94 H9-4.2.5 (Fig 1). In comparison, NA genomic diversity remained relatively stable. However, this could be an artefact caused by the limited number of Cambodian A(H9N2) NA sequences available prior to 2015. The HA, NA and internal gene phylogenies show that these viruses possibly arose from AIVs detected elsewhere in Asia, and are closely related to AIVs concurrently detected in Vietnam. Similar observations between endemically circulating Cambodian and Vietnamese A(H5N1) AIVs also exist. Transmission of A(H5) and A(H9) AIVs between the two countries is likely to be facilitated by cross-border movement of domestic poultry.

Transmission by migration of wild birds may also play a minor role in viral diversity in Cambodia. A single A(H9N2) isolate, designated A/chicken/Cambodia/a77W22M1/2016, clustered separately from other Cambodian viruses, possibly representing a separate A(H9) introduction via migration of wild birds along the Eastern Asia/Australasian flyway. This virus clustered with A(H9N2) AIVs detected in poultry from Vietnam between 2014 and 2018, Japan in 2016 and with Indonesian A(H9N2) AIVs detected from 2016 to 2018. The first detection of A(H9N2) in Indonesia occurred during AIV outbreaks in 2016 and was associated with increased flock mortality and an 18% reduction in the egg production of layers [90]. Further surveillance for A(H9) AIVs in domestic poultry and wild birds is vital to understand the dynamics of A(H9) transmission between these countries.

Since 2013, A(H9) genotype S viruses have been the most prevalent AIV lineage detected in Chinese chickens [18]. The enhanced fitness of genotype S in chickens is partly attributable to the acquisition of G1/97-like MP and PB2 genes. These genes increase AIV polymerase activity, replicative capacity, and virulence of AIVs in chickens [91,92]. Both Cambodian genotype P and V viruses contained G1/97-like matrix proteins, but only genotype V also has a G1/97-like PB2. Interestingly, the prevalence of genotype V in Cambodian poultry increased from 2015 to 2016; however, this finding may be an artefact of limited sampling performed in 2016 compared to 2015.

The internal genomic cassette of genotype S viruses have been repeatedly donated to zoonotic AIVs such as A(H5N6), A(H7N9) and A(H10N8) [18–21]. In Cambodia, genotype S viruses have not been detected, although A(H9N2) viruses have multiple segments that cluster with genotype S. This finding is unsurprising considering that the genotype V viruses, originally described in China 2014, arose from the reassortment of genotype S viruses that acquired G9/97 NA genes [1,49]. Interestingly, the Indonesian-like isolate from 2016 (A/chicken/Cambodia/a77W22M1/2016) contained PB2, PA, MP and NS genes that clustered closely with zoonotic A(H7N9), A(H9N2) and/or A(H10N8) AIVs (nucleotide sequence identity >98%). Consequently, introduction of new clades and continual reassortment combined with the endemicity of A(H9N2) viruses in Cambodian poultry raises concerns about the emergence of novel zoonotic AIVs.

Molecular analyses indicate the Cambodian A(H9N2) AIVs were LPAI viruses with multiple molecular markers associated with adaptation to mammalian species. Cambodian A(H9N2) viruses contained multiple HA markers associated with increased binding to “human-like” α2,6-linked sialic acid residues (Table 1). One well known HA substitution, Q226L, significantly increases the binding of A(H9) AIVs to α2,6-linked sialic acids and enhances AIV transmission in ferrets [30,79]. Between 2013 to 2016, the prevalence of Q226L in Cambodian A(H9N2) viruses increased from 50% to 100% similar to BJ/94 and G1/97 lineage A(H9) viruses in China, Vietnam and the Middle East [1,30,93]. The rising global prevalence of Q226L is concerning considering the enhancement of zoonotic potential. LBM studies performed in Cambodia in 2013 showed 1.8% of LBM workers had seroconverted to the A(H9) subtype [24]. Further work is needed to see if increased subclinical, zoonotic transmission has occurred with this change in receptor specificity.

Other substitutions identified in internal proteins of Cambodian A(H9N2) viruses have also been associated with increased AIV replication and virulence in mammals (Table 1). While major molecular markers of AIV adaptation to mammals in PB2, namely E627K and D701N, were not identified, a small number of isolates contained A588V (n = 1) and Q591K (n = 3) substitutions that partially compensate for the absence of E627K [61,63]. In particular, A588V increases the polymerase activity and replication of A(H7N9) and A(H9N2) viruses in mammalian and avian cell lines, consequently increasing AIV virulence in mammalian models [61]. Since 2013, the number of A(H9N2) and zoonotic A(H7N9) viruses with A588V substitutions has increased significantly at the global level [61].

Candidate A(H9) vaccine viruses are selected as part of pandemic preparedness plans [94]. The Cambodian A(H9N2) viruses have the highest nucleotide identity to A/Hong Kong/308/2014 (94.1% to 95.1%), though they are more similar to G9/97 at HA antigenic sites. The most appropriate CVV to protect against Cambodian A(H9) viruses would need to be determined experimentally. Additionally, vaccination of poultry, in combination with biosecurity programs, can effectively decrease AIV circulation in poultry flocks and therefore decrease human exposure. However, the most commonly used commercially available A(H9) poultry vaccines in Asia, are produced from viruses detected in the 1990s [95]. As a result, A(H9) poultry vaccine effectiveness continually decreases due to ongoing antigenic drift [95]. No official poultry vaccination against AIVs occurs in Cambodia. Cambodian viruses have the highest sequence identity to SS/94 (91.8% to 90.5% nucleotide identity), though the HA genes differ at six to nine HA antigenic sites. Therefore, it is possible that this variation could reduce vaccine effectiveness, but further experimental work is warranted.

Assessing the effectiveness of available antivirals is vital for combating zoonotic AIVs as part of influenza pandemic preparedness strategies [94]. Molecular data from Cambodian A(H9N2) viruses indicates that 41% of isolates contained the S31N mutation in the M2 protein, indicative of resistance to adamantanes [84,96]. Furthermore, reassortment between these A(H9N2) viruses and endemically circulating A(H5N1) in Cambodia has resulted in a A(H5N1) clade 2.3.2.1c virus with the MP gene of A(H9) origin containing the M2 S31N marker [97]. As this A(H5) clade does not typically contain markers of adamantine resistance, this is one example of a reassortment event increasing AIV risk [1,30]. However, adamantanes are not recommended for treating influenza as resistance is widespread in both seasonal and other potentially zoonotic AIVs [98]. All Cambodian viruses tested remain susceptible to the first line NAI antivirals: oseltamivir, zanamivir, laninamivir and peramivir.

In summary, Cambodian A(H9N2) AIVs isolated from domestic poultry in LBMs between 2015 and 2016 belonged to the BJ/94 H9-4.2.5 lineage. They were closely related to viruses identified in surrounding countries, suggesting frequent circulation of these viruses possibly through cross-border movement of live poultry. Wild birds may also play a role in A(H9) transmission. The Cambodian A(H9N2) viruses contained multiple genomic segments clustering with genotype S lineage AIVs, with a small number close to zoonotic A(H7N9), A(H9N2) and A(H10N8) viruses. Additionally, all Cambodian A(H9N2) viruses had molecular markers associated with viral adaptation to mammalian species, raising further concerns about their increased zoonotic potential. However, these viruses remained susceptible to therapeutic and prophylactic prevention strategies utilizing NAIs. Overall, the continued endemicity and evolution of A(H9N2) AIVs in Cambodian domestic poultry underlines the need for continued, vigilant surveillance analysis of AIVs within the country.

## Supporting information

S1 Fig Map of sample collection sites in Cambodia from 2015 to 2016. Samples were collected from two LBMs during this period, Orussey market (Phnom Penh) and Takeo market (Takeo), indicated by red dots. The map was produced with QGIS v2.18.4 using public domain data obtained from Natural Earth (http://www.naturalearthdata.com/).
(TIF)

S2 Fig Bayesian maximum clade credibility (MCC) phylogenetic trees of Cambodian A(H9N2) isolates identified from 2013 to 2016 produced from the a) HA and b) NA genomic segments. Cambodian AIVs are coloured based on the year of detection: 2013 is shown in orange, 2015 in green and 2016 in blue. Trees were produced using BEAST v1.84 with GTR + Γ and SRD06 nucleotide substitution model. The nodes are labelled with the proposed TMRCA and node bars show corresponding 95% HPD ranges. Branch lengths are time-proportional and the time scale is shown on the x-axis.
(PDF)

S3 Fig Bayesian skyride analysis of Cambodian A(H9N2) genetic diversity. Genetic diversity of the HA and NA A(H9N2) genes was estimated using the Gaussian Markov Random Field (GMRF) model. The x-axis measures time in years and the y-axis is an estimate of genetic diversity calculated from Neτ (effective population size and the generation length in years) shown in log scale. The median estimate of genetic diversity over time is shown as a solid black line and the purple shading represents the 95% HPD intervals.
(TIF)

S4 Fig Maximum likelihood phylogenetic trees for all genomic segments of Cambodian A(H9N2) viruses isolated from 2015 and 2016. **a) PB2 b) PB1 c) PA d) NP e) MP and f) NS.** Trees were generated with IQ-Tree using the GTR+ I + Γ model with 1,000 ultrafast boostrap replicates. Cambodian viruses are coloured based on the year of detection: 2015 is light blue and 2016 dark blue. AIVs identified in humans are coloured red, and G57 lineage viruses (as defined by Pu et al., 2015) are pink. Candidate vaccine viruses are indicated by an asterisks (*) next to the taxa name. Viruses from A(H9) reference lineages are shown in bold and the lineage Cambodian A(H9N2) viruses fall under is indicated on the right hand side of the tree. Bootstrap values of 70 or greater are displayed on branches. The scale bar indicates number of nucleotide substitutions per site.
(PDF)

S1 Table Summary of Cambodian A(H9N2) viruses identified in 2015 and 2016 that were analysed as part of this study with sample collection details, genotyping information and sequence accession numbers listed. (XLSX)

S2 Table List of AIVs used to analyse phylogenetic congruence. (XLSX)

S3 Table a-h Molecular analysis of the eight genomic segments for the Cambodian A(H9N2) isolates identified between 2015 and 2016. Data for each segment, as well as any associated accessory proteins is listed: a) PB2, b) PB1, c) PA, d) HA, e) NP, f) NA, g) MP and h) NS
(XLSX)

S4 Table Analysis of HA antigenic sites in Cambodian A(H9N2) AIVs compared to human and poultry vaccine viruses. (XLSX)

S5 Table N-glycosylation sites in the HA and NA proteins predicted for Cambodian A(H9N2) isolates identified between 2015 and 2016. (XLSX)

S6 Table Selection pressure analysis of the Cambodian A(H9N2) genes using FEL, FUBAR, MEME and SLAC. (XLSX)

S7 Table Susceptibility of Cambodia A(H9N2) isolates to a panel of four neuraminidase inhibitors: oseltamivir, zanamivir, laninamivir and peramivir. (XLSX)

## References

[1] Liu Y-F, Lai H-Z, Li L, Liu Y-P, Zhang W-Y, Gao R, et al Endemic Variation of H9N2 Avian Influenza Virus in China. Avian Dis 2016;60:817–25. 10.1637/11452-061616-Reg 27902899

[2] Nagy A, Mettenleiter TC, Abdelwhab EM. A brief summary of the epidemiology and genetic relatedness of avian influenza H9N2 virus in birds and mammals in the Middle East and North Africa. Epidemiol Infect 2017;145:3320–33. 10.1017/S0950268817002576 29168447 PMC9148743

[3] Li C, Yu K, Tian G, Yu D, Liu L, Jing B, et al Evolution of H9N2 influenza viruses from domestic poultry in Mainland China. Virology 2005;340:70–83. 10.1016/j.virol.2005.06.025 16026813

[4] Gu M, Xu L, Wang X, Liu X. Current situation of H9N2 subtype avian influenza in China. Vet Res 2017;48:49 10.1186/s13567-017-0453-2 28915920 PMC5603032

[5] Qi X, Tan D, Wu C, Tang C, Li T, Han X, et al Deterioration of eggshell quality in laying hens experimentally infected with H9N2 avian influenza virus. Vet Res 2016;47 10.1186/s13567-016-0332-2 26915662 PMC4766683

[6] Naeem K, Naurin M, Rashid S, Bano S. Seroprevalence of avian influenza virus and its relationship with increased mortality and decreased egg production. Avian Pathol 2003;32:283–7. 10.1080/0307945031000097886. 12850918

[7] Kishida N, Sakoda Y, Eto M, Sunaga Y, Kida H. Co-infection of Staphylococcus aureus or Haemophilus paragallinarum exacerbates H9N2 influenza A virus infection in chickens. Arch Virol 2004;149:2095–2104. 10.1007/s00705-004-0372-1 15503199

[8] Azizpour A, Goudarzi H, Charkhkar S, Momayez R, Hablolvarid M, others. Experimental study on tissue tropism and dissemination of H9N2 avian influenza virus and Ornithobacterium rhinotracheale co-infection in SPF chickens. JAPS J Anim Plant Sci 2014;24:1655–62.

[9] Umar S, Guerin JL, Ducatez MF. Low Pathogenic Avian Influenza and Coinfecting Pathogens: A Review of Experimental Infections in Avian Models. Avian Dis 2017;61:3–15. 10.1637/11514-101316-Review 28301244

[10] Nili H, Asasi K. Avian Influenza (H9N2) Outbreak in Iran. Avian Dis 2003;47:828–31. 10.1637/0005-2086-47.s3.828 14575072

[11] Nili H, Asasi K. Natural cases and an experimental study of H9N2 avian influenza in commercial broiler chickens of Iran. Avian Pathol 2002;31:247–52. 10.1080/03079450220136567 12396348

[12] WHO. Influenza at the human-animal interface: Summary and assessment, 2 November to 13 December 2018. 2018.

[13] Peacock TP, James J, Sealy JE, Iqbal M. A Global Perspective on H9N2 Avian Influenza Virus. Viruses 2019;11:620 10.3390/v11070620. PMC6669617 31284485

[14] World Health Organisation. Influenza at the human-animal interface Summary and assessment, 20 July to 3 October 2016. 2016.

[15] Guo YJ, Krauss S, Senne DA, Mo IP, Lo KS, Xiong XP, et al Characterization of the Pathogenicity of Members of the Newly Established H9N2 Influenza Virus Lineages in Asia. Virology 2000;267:279–88. 10.1006/viro.1999.0115 10662623

[16] Huang Y, Li X, Zhang H, Chen B, Jiang Y, Yang L, et al Human infection with an avian influenza A (H9N2) virus in the middle region of China: Human Infection With an Avian Influenza A (H9N2) Virus. J Med Virol 2015;87:1641–8. 10.1002/jmv.24231 25965534

[17] Peiris M, Yuen KY, Leung CW, Chan KH, Ip PLS, Lai RWM, et al Human infection with influenza H9N2. The Lancet 1999;354:916–917. 10.1016/s0140-6736(99)03311-5 10489954

[18] Pu J, Wang S, Yin Y, Zhang G, Carter RA, Wang J, et al Evolution of the H9N2 influenza genotype that facilitated the genesis of the novel H7N9 virus. Proc Natl Acad Sci 2015;112:548–53. 10.1073/pnas.1422456112 25548189 PMC4299237

[19] Shen Y-Y, Ke C-W, Li Q, Yuan R-Y, Xiang D, Jia W-X, et al Influenza A(H5N6) Viruses in Humans, Guangdong, China, 2015. Emerg Infect Dis 2016;22:3. 10.3201/eid2208.160146 PMC4982152 27331418

[20] Zhang T, Bi Y, Tian H, Li X, Liu D, Wu Y, et al Human Infection with Influenza Virus A(H10N8) from Live Poultry Markets, China, 2014. Emerg Infect Dis 2014;20 10.3201/eid2012.140911. PMC4257803 25425075

[21] Liu D, Shi W, Shi Y, Wang D, Xiao H, Li W, et al Origin and diversity of novel avian influenza A H7N9 viruses causing human infection: phylogenetic, structural, and coalescent analyses. The Lancet 2013;381:1926–32. 10.1016/S0140-6736(13)60938-1. 23643111

[22] Gu M, Chen H, Li Q, Huang J, Zhao M, Gu X, et al Enzootic genotype S of H9N2 avian influenza viruses donates internal genes to emerging zoonotic influenza viruses in China. Vet Microbiol 2014;174:309–15. 10.1016/j.vetmic.2014.09.029 25457363

[23] Guan Y, Shortridge KF, Krauss S, Webster RG. Molecular characterization of H9N2 influenza viruses: were they the donors of the “internal” genes of H5N1 viruses in Hong Kong? Proc Natl Acad Sci 1999;96:9363–9367. 10.1073/pnas.96.16.9363 10430948 PMC17788

[24] Horm SV, Tarantola A, Rith S, Ly S, Gambaretti J, Duong V, et al Intense circulation of A/H5N1 and other avian influenza viruses in Cambodian live-bird markets with serological evidence of sub-clinical human infections. Emerg Microbes Infect 2016;5:e70 10.1038/emi.2016.69 27436362 PMC5141262

[25] Horm SV, Sorn S, Allal L, Buchy P. Influenza A(H5N1) Virus Surveillance at Live Poultry Markets, Cambodia, 2011. Emerg Infect Dis 2013;19:305–8. 10.3201/eid1902.121201 23347451 PMC3559060

[26] Vijaykrishna D, Deng Y-M, Grau ML, Kay M, Suttie A, Horwood PF, et al Emergence of Influenza A(H7N4) Virus, Cambodia. Emerg Infect Dis 2019;25 10.3201/eid2510.190506. PMC6759271 31310233

[27] Suttie A, Yann S, Y P, Tum S, Deng Y-M, Hul V, et al Detection of Low Pathogenicity Influenza A(H7N3) Virus during Duck Mortality Event, Cambodia, 2017. Emerg Infect Dis 2018;24:1103–7. 10.3201/eid2406.172099 29774842 PMC6004859

[28] Horwood PF, Horm SV, Suttie A, Thet S, Phalla Y, Rith S, et al Co-circulation of Influenza A H5, H7, and H9 Viruses and Co-infected Poultry in Live Bird Markets, Cambodia. Emerg Infect Dis 2018;24 10.3201/eid2402.171360. PMC5782910 29350140

[29] Karlsson EA, Horm SV, Tok S, Tum S, Kalpravidh W, Claes F, et al Avian influenza virus detection, temporality and co-infection in poultry in Cambodian border provinces, 2017–2018. Emerg Microbes Infect 2019;8:637–9. 10.1080/22221751.2019.1604085 30999819 PMC6493305

[30] Thuy DM, Peacock TP, Bich VTN, Fabrizio T, Hoang DN, Tho ND, et al Prevalence and diversity of H9N2 avian influenza in chickens of Northern Vietnam, 2014. Infect Genet Evol 2016;44:530–40. 10.1016/j.meegid.2016.06.038 27340015 PMC5036934

[31] Kim Y, Biswas PK, Giasuddin M, Hasan M, Mahmud R, Chang Y-M, et al Prevalence of Avian Influenza A(H5) and A(H9) Viruses in Live Bird Markets, Bangladesh. Emerg Infect Dis 2018;24:2309–16. 10.3201/eid2412.180879 30457545 PMC6256373

[32] Luo S, Xie Z, Xie Z, Xie L, Huang L, Huang J, et al Surveillance of Live Poultry Markets for Low Pathogenic Avian Influenza Viruses in Guangxi Province, Southern China, from 2012–2015. Sci Rep 2017;7 10.1038/s41598-017-17740-0. PMC5730573 29242521

[33] Van Kerkhove MD, Vong S, Guitian J, Holl D, Mangtani P, San S, et al Poultry movement networks in Cambodia: Implications for surveillance and control of highly pathogenic avian influenza (HPAI/H5N1). Vaccine 2009;27:6345–52. 10.1016/j.vaccine.2009.05.004 19840671

[34] Horm SV, Gutiérrez RA, Sorn S, Buchy P. Environment: a potential source of animal and human infection with influenza A (H5N1) virus: Influenza A (H5N1) virus in the environment. Influenza Other Respir Viruses 2012;6:442–8. 10.1111/j.1750-2659.2012.00338.x 22340982 PMC4986626

[35] Zhou B, Wentworth DE. Influenza A Virus Molecular Virology Techniques In: Kawaoka Y, Neumann G, editors. Influenza Virus, vol. 865, Totowa, NJ: Humana Press; 2012, p. 175–92. 10.1007/978-1-61779-621-0_11. 22528160

[36] Hoffmann E, Stech J, Guan Y, Webster RG, Perez DR. Universal primer set for the full-length amplification of all influenza A viruses. Arch Virol 2001;146:2275–2289. 10.1007/s007050170002 11811679

[37] Shu Y, McCauley J. GISAID: Global initiative on sharing all influenza data–from vision to reality. Eurosurveillance 2017;22. 10.2807/1560-7917.ES.2017.22.13.30494 PMC5388101 28382917

[38] Benson DA, Cavanaugh M, Clark K, Karsch-Mizrachi I, Lipman DJ, Ostell J, et al GenBank. Nucleic Acids Res 2013;41:D36–42. 10.1093/nar/gks1195 23193287 PMC3531190

[39] Squires RB, Noronha J, Hunt V, García-Sastre A, Macken C, Baumgarth N, et al Influenza Research Database: an integrated bioinformatics resource for influenza research and surveillance: Influenza Research Database. Influenza Other Respir Viruses 2012;6:404–16. 10.1111/j.1750-2659.2011.00331.x 22260278 PMC3345175

[40] Katoh K, Standley DM. MAFFT Multiple Sequence Alignment Software Version 7: Improvements in Performance and Usability. Mol Biol Evol 2013;30:772–80. 10.1093/molbev/mst010 23329690 PMC3603318

[41] Nguyen L-T, Schmidt HA, von Haeseler A, Minh BQ. IQ-TREE: a fast and effective stochastic algorithm for estimating maximum-likelihood phylogenies. Mol Biol Evol 2015;32:268–274. 10.1093/molbev/msu300 25371430 PMC4271533

[42] Minh BQ, Nguyen MAT, von Haeseler A. Ultrafast Approximation for Phylogenetic Bootstrap. Mol Biol Evol 2013;30:1188–95. 10.1093/molbev/mst024 23418397 PMC3670741

[43] Tavaré S. Some probabilistic and statistical problems in the analysis of DNA sequences. Lect Math Life Sci 1986;17:57–86.

[44] Drummond AJ, Suchard MA, Xie D, Rambaut A. Bayesian Phylogenetics with BEAUti and the BEAST 1.7. Mol Biol Evol 2012;29:1969–73. 10.1093/molbev/mss075 22367748 PMC3408070

[45] Shapiro B, Rambaut A, Drummond AJ. Choosing Appropriate Substitution Models for the Phylogenetic Analysis of Protein-Coding Sequences. Mol Biol Evol 2006;23:7–9. 10.1093/molbev/msj021 16177232

[46] Drummond AJ, Ho SY, Phillips MJ, Rambaut A. Relaxed phylogenetics and dating with confidence. PLoS Biol 2006;4:e88 10.1371/journal.pbio.0040088 16683862 PMC1395354

[47] Minin VN, Bloomquist EW, Suchard MA. Smooth Skyride through a Rough Skyline: Bayesian Coalescent-Based Inference of Population Dynamics. Mol Biol Evol 2008;25:1459–71. 10.1093/molbev/msn090 18408232 PMC3302198

[48] Andrew Rambaut. Molecular evolution, phylogenetics and epidemiology: FigTree 2016 http://tree.bio.ed.ac.uk/software/figtree/.

[49] Gu M, Xu L, Wang X, Liu X. Current situation of H9N2 subtype avian influenza in China. Vet Res 2017;48:49 10.1186/s13567-017-0453-2 28915920 PMC5603032

[50] Suttie A, Deng Y-M, Greenhill AR, Dussart P, Horwood PF, Karlsson EA. Inventory of molecular markers affecting biological characteristics of avian influenza A viruses. Virus Genes 2019 10.1007/s11262-019-01700-z. PMC6831541 31428925

[51] Gupta R, Jung E, Brunak S. Prediction of N-glycosylation sites in human proteins. 2004. Ref Type Unpubl Work 2016.

[52] Pond SLK, Muse SV. HyPhy: hypothesis testing using phylogenies Stat. Methods Mol. Evol., Springer; 2005, p. 125–181.

[53] Weaver S, Shank SD, Spielman SJ, Li M, Muse SV, Kosakovsky Pond SL. Datamonkey 2.0: a modern web application for characterizing selective and other evolutionary processes. Mol Biol Evol 2018;35:773–777. 10.1093/molbev/msx335 29301006 PMC5850112

[54] Murrell B, Moola S, Mabona A, Weighill T, Sheward D, Kosakovsky Pond SL, et al FUBAR: a fast, unconstrained bayesian approximation for inferring selection. Mol Biol Evol 2013;30:1196–1205. 10.1093/molbev/mst030 23420840 PMC3670733

[55] Murrell B, Wertheim JO, Moola S, Weighill T, Scheffler K, Pond SLK. Detecting individual sites subject to episodic diversifying selection. PLoS Genet 2012;8:e1002764 10.1371/journal.pgen.1002764 22807683 PMC3395634

[56] Kosakovsky Pond SL, Frost SD. Not so different after all: a comparison of methods for detecting amino acid sites under selection. Mol Biol Evol 2005;22:1208–1222. 10.1093/molbev/msi105 15703242

[57] Leang S-K, Hurt AC. Fluorescence-based Neuraminidase Inhibition Assay to Assess the Susceptibility of Influenza Viruses to The Neuraminidase Inhibitor Class of Antivirals. J Vis Exp 2017 10.3791/55570. PMC5564701 28448045

[58] Jiang W, Liu S, Hou G, Li J, Zhuang Q, Wang S, et al Chinese and Global Distribution of H9 Subtype Avian Influenza Viruses. PLoS ONE 2012;7:e52671 10.1371/journal.pone.0052671 23285143 PMC3528714

[59] Huang Y, Hu B, Wen X, Cao S, Gavrilov BK, Du Q, et al Diversified reassortant H9N2 avian influenza viruses in chicken flocks in northern and eastern China. Virus Res 2010;151:26–32. 10.1016/j.virusres.2010.03.010 20347894

[60] Zhu R, Xu D, Yang X, Zhang J, Wang S, Shi H, et al Genetic and biological characterization of H9N2 avian influenza viruses isolated in China from 2011 to 2014. PLOS ONE 2018;13:e0199260 10.1371/journal.pone.0199260 29969454 PMC6029760

[61] Xiao C, Ma W, Sun N, Huang L, Li Y, Zeng Z, et al PB2-588 V promotes the mammalian adaptation of H10N8, H7N9 and H9N2 avian influenza viruses. Sci Rep 2016;6 10.1038/srep19474. PMC4726052 26782141

[62] Hu M, Yuan S, Zhang K, Singh K, Ma Q, Zhou J, et al PB2 substitutions V598T/I increase the virulence of H7N9 influenza A virus in mammals. Virology 2017;501:92–101. 10.1016/j.virol.2016.11.008 27889648

[63] Wang C, Lee HHY, Yang ZF, Mok CKP, Zhang Z. PB2-Q591K Mutation Determines the Pathogenicity of Avian H9N2 Influenza Viruses for Mammalian Species. PLOS ONE 2016;11:e0162163 10.1371/journal.pone.0162163 27684944 PMC5042486

[64] Sang X, Wang A, Chai T, He X, Ding J, Gao X, et al Rapid emergence of a PB2-E627K substitution confers a virulent phenotype to an H9N2 avian influenza virus during adoption in mice. Arch Virol 2015;160:1267–77. 10.1007/s00705-015-2383-5 25782865

[65] Sediri H, Thiele S, Schwalm F, Gabriel G, Klenk H-D. PB2 subunit of avian influenza virus subtype H9N2: a pandemic risk factor. J Gen Virol 2016;97:39–48. 10.1099/jgv.0.000333 26560088

[66] Feng X, Wang Z, Shi J, Deng G, Kong H, Tao S, et al Glycine at Position 622 in PB1 Contributes to the Virulence of H5N1 Avian Influenza Virus in Mice. J Virol 2016;90:1872–9. 10.1128/JVI.02387-15 26656683 PMC4733975

[67] James J, Howard W, Iqbal M, Nair VK, Barclay WS, Shelton H. Influenza A virus PB1-F2 protein prolongs viral shedding in chickens lengthening the transmission window. J Gen Virol 2016;97:2516–27. 10.1099/jgv.0.000584 27558742 PMC5078828

[68] Hu M, Chu H, Zhang K, Singh K, Li C, Yuan S, et al Amino acid substitutions V63I or A37S/I61T/V63I/V100A in the PA N-terminal domain increase the virulence of H7N7 influenza A virus. Sci Rep 2016;6:37800 10.1038/srep37800 27886255 PMC5122915

[69] Xu G, Zhang X, Gao W, Wang C, Wang J, Sun H, et al Prevailing PA Mutation K356R in Avian Influenza H9N2 Virus Increases Mammalian Replication and Pathogenicity. J Virol 2016;90:8105–14. 10.1128/JVI.00883-16 27384648 PMC5008101

[70] Gao H, Liu J, Kong W, Sun H, Pu J, Chang K-C, et al PA-X is a virulence factor in avian H9N2 influenza virus. J Gen Virol 2015;96:2587–94. 10.1099/jgv.0.000232 26296365

[71] Baron J, Tarnow C, Mayoli-Nüssle D, Schilling E, Meyer D, Hammami M, et al Matriptase, HAT, and TMPRSS2 activate the hemagglutinin of H9N2 influenza A viruses. J Virol 2013;87:1811–1820. 10.1128/JVI.02320-12 23192872 PMC3554176

[72] Gohrbandt S, Veits J, Breithaupt A, Hundt J, Teifke JP, Stech O, et al H9 avian influenza reassortant with engineered polybasic cleavage site displays a highly pathogenic phenotype in chicken. J Gen Virol 2011;92:1843–53. 10.1099/vir.0.031591-0 21525207

[73] Su Y, Yang H-Y, Zhang B-J, Jia H-L, Tien P. Analysis of a point mutation in H5N1 avian influenza virus hemagglutinin in relation to virus entry into live mammalian cells. Arch Virol 2008;153:2253–61. 10.1007/s00705-008-0255-y 19020946

[74] Auewarakul P, Suptawiwat O, Kongchanagul A, Sangma C, Suzuki Y, Ungchusak K, et al An avian influenza H5N1 virus that binds to a human-type receptor. J Virol 2007;81:9950–5. 10.1128/JVI.00468-07 17626098 PMC2045398

[75] Wang W, Lu B, Zhou H, Suguitan AL, Cheng X, Subbarao K, et al Glycosylation at 158N of the Hemagglutinin Protein and Receptor Binding Specificity Synergistically Affect the Antigenicity and Immunogenicity of a Live Attenuated H5N1 A/Vietnam/1203/2004 Vaccine Virus in Ferrets. J Virol 2010;84:6570–7. 10.1128/JVI.00221-10 20427525 PMC2903256

[76] Teng Q, Xu D, Shen W, Liu Q, Rong G, Li X, et al A Single Mutation at Position 190 in Hemagglutinin Enhances Binding Affinity for Human Type Sialic Acid Receptor and Replication of H9N2 Avian Influenza Virus in Mice. J Virol 2016;90:9806–25. 10.1128/JVI.01141-16 27558420 PMC5068531

[77] Yamada S, Suzuki Y, Suzuki T, Le MQ, Nidom CA, Sakai-Tagawa Y, et al Haemagglutinin mutations responsible for the binding of H5N1 influenza A viruses to human-type receptors. Nature 2006;444:378–82. 10.1038/nature05264 17108965

[78] Wan H, Sorrell EM, Song H, Hossain MJ, Ramirez-Nieto G, Monne I, et al Replication and Transmission of H9N2 Influenza Viruses in Ferrets: Evaluation of Pandemic Potential. PLoS ONE 2008;3:e2923 10.1371/journal.pone.0002923 18698430 PMC2500216

[79] Wan H, Perez DR. Amino Acid 226 in the Hemagglutinin of H9N2 Influenza Viruses Determines Cell Tropism and Replication in Human Airway Epithelial Cells. J Virol 2007;81:5181–91. 10.1128/JVI.02827-06 17344280 PMC1900221

[80] Tada T, Suzuki K, Sakurai Y, Kubo M, Okada H, Itoh T, et al NP body domain and PB2 contribute to increased virulence of H5N1 highly pathogenic avian influenza viruses in chickens. J Virol 2011;85:1834–46. 10.1128/JVI.01648-10 21123376 PMC3028894

[81] Nao N, Kajihara M, Manzoor R, Maruyama J, Yoshida R, Muramatsu M, et al A Single Amino Acid in the M1 Protein Responsible for the Different Pathogenic Potentials of H5N1 Highly Pathogenic Avian Influenza Virus Strains. PloS One 2015;10:e0137989 10.1371/journal.pone.0137989 26368015 PMC4569272

[82] Smeenk CA, Wright KE, Burns BF, Thaker AJ, Brown EG. Mutations in the hemagglutinin and matrix genes of a virulent influenza virus variant, A/FM/1/47-MA, control different stages in pathogenesis. Virus Res 1996;44:79–95. 10.1016/0168-1702(96)01329-9 8879138

[83] Brown EG, Bailly JE. Genetic analysis of mouse-adapted influenza A virus identifies roles for the NA, PB1, and PB2 genes in virulence. Virus Res 1999;61:63–76. 10.1016/s0168-1702(99)00027-1 10426210

[84] Ilyushina NA, Govorkova EA, Webster RG. Detection of amantadine-resistant variants among avian influenza viruses isolated in North America and Asia. Virology 2005;341:102–6. 10.1016/j.virol.2005.07.003 16081121

[85] Jiao P, Tian G, Li Y, Deng G, Jiang Y, Liu C, et al A Single-Amino-Acid Substitution in the NS1 Protein Changes the Pathogenicity of H5N1 Avian Influenza Viruses in Mice. J Virol 2008;82:1146–54. 10.1128/JVI.01698-07 18032512 PMC2224464

[86] Li Z, Jiang Y, Jiao P, Wang A, Zhao F, Tian G, et al The NS1 Gene Contributes to the Virulence of H5N1 Avian Influenza Viruses. J Virol 2006;80:11115–23. 10.1128/JVI.00993-06 16971424 PMC1642184

[87] Peacock TP, Harvey W, Sadeyen J-R, Reeve R, Iqbal M. The molecular basis of antigenic variation among A(H9N2) avian influenza viruses. BioRxiv 2018 10.1101/312967. PMC6220119 30401826

[88] Noshi T, Kitano M, Taniguchi K, Yamamoto A, Omoto S, Baba K, et al In vitro characterization of baloxavir acid, a first-in-class cap-dependent endonuclease inhibitor of the influenza virus polymerase PA subunit. Antiviral Res 2018;160:109–17. 10.1016/j.antiviral.2018.10.008 30316915

[89] Omoto S, Speranzini V, Hashimoto T, Noshi T, Yamaguchi H, Kawai M, et al Characterization of influenza virus variants induced by treatment with the endonuclease inhibitor baloxavir marboxil. Sci Rep 2018;8:9633 10.1038/s41598-018-27890-4 29941893 PMC6018108

[90] Jonas M, Sahesti A, Murwijati T, Lestariningsih CL, Irine I, Ayesda CS, et al Identification of avian influenza virus subtype H9N2 in chicken farms in Indonesia. Prev Vet Med 2018;159:99–105. 10.1016/j.prevetmed.2018.09.003 30314797

[91] Pu J, Sun H, Qu Y, Wang C, Gao W, Zhu J, et al M Gene Reassortment in H9N2 Influenza Virus Promotes Early Infection and Replication: Contribution to Rising Virus Prevalence in Chickens in China. J Virol 2017;91 10.1128/JVI.02055-16. PMC5375663 28148803

[92] Hao X, Wang X, Hu J, Gu M, Wang J, Deng Y, et al The PB2 and M genes of genotype S H9N2 virus contribute to the enhanced fitness of H5Nx and H7N9 avian influenza viruses in chickens. Virology 2019 10.1016/j.virol.2019.07.001. 31325836

[93] Chrzastek K, Lee D, Gharaibeh S, Zsak A, Kapczynski DR. Characterization of H9N2 avian influenza viruses from the Middle East demonstrates heterogeneity at amino acid position 226 in the hemagglutinin and potential for transmission to mammals. Virology 2018;518:195–201. 10.1016/j.virol.2018.02.016 29524835

[94] World Health Organization. Influenza: Candidate vaccine viruses for A(H9N2) 2019. https://www.who.int/influenza/vaccines/virus/candidates_reagents/a_h9n2/en/.

[95] Xu C, Ye H, Qiu W, Lin H, Chen Y, Zhang H, et al Phylogenetic classification of hemagglutinin gene of H9N2 avian influenza viruses isolated in China during 2012–2016 and evaluation of selected candidate vaccine strains. Poult Sci 2018;97:3023–30. 10.3382/ps/pey154 29931183

[96] Belshe RB, Smith MH, Hall CB, Betts R, Hay AJ. Genetic basis of resistance to rimantadine emerging during treatment of influenza virus infection. J Virol 1988;62:1508–1512. 3282079 10.1128/jvi.62.5.1508-1512.1988 PMC253174

[97] Suttie A, Karlsson EA, Deng Y-M, Horm SV, Yann S, Tok S, et al Influenza A(H5N1) viruses with A(H9N2) single gene (matrix or PB1) reassortment isolated from Cambodian live bird markets. Virology 2018;523:22–6. 10.1016/j.virol.2018.07.028 30075357

[98] Dong G, Peng C, Luo J, Wang C, Han L, Wu B, et al Adamantane-Resistant Influenza A Viruses in the World (1902–2013): Frequency and Distribution of M2 Gene Mutations. PLOS ONE 2015;10:e0119115 10.1371/journal.pone.0119115 25768797 PMC4358984

