# Supplementary figures and images for: The evolution and genetic diversity of avian influenza A(H9N2) viruses in Cambodia, 2015 – 2016
Source: PLoS One. 2019 Dec 9;14(12):e0225428. doi: 10.1371/journal.pone.0225428 (PMC6901181; doi:10.1371/journal.pone.0225428)

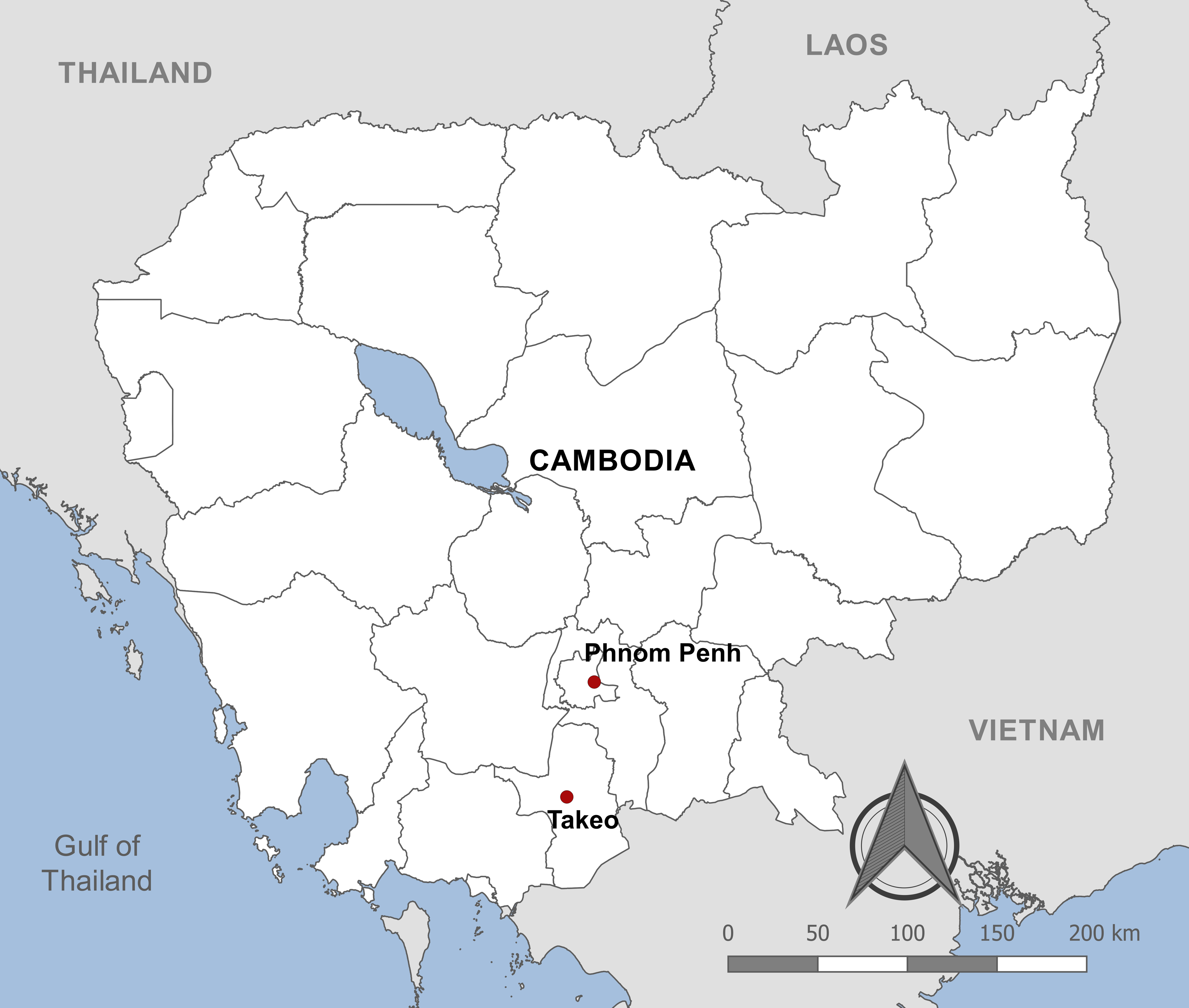

Supplement: S1 Fig — Samples were collected from two LBMs during this period, Orussey market (Phnom Penh) and Takeo market (Takeo), indicated by red dots. The map was produced with QGIS v2.18.4 using public domain data obtained from Natural Earth (http://www.naturalearthdata.com/). (TIF) [file pone.0225428.s001.tif]

S2a) HA

Key

- Cambodian 2013
- Cambodian 2015
- Cambodian 2016

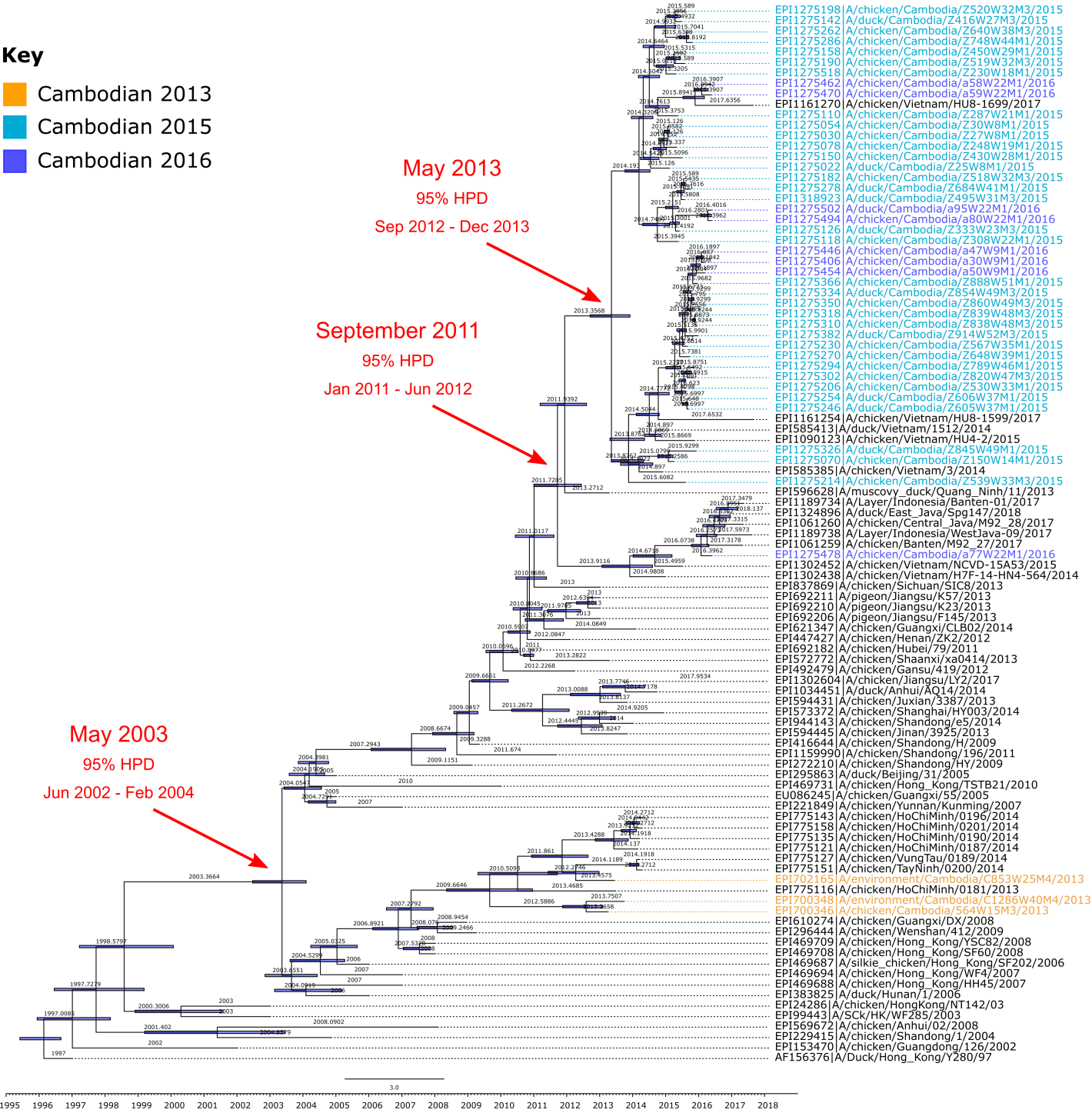

S2b) NA

Key

- Cambodian 2013
- Cambodian 2015
- Cambodian 2016

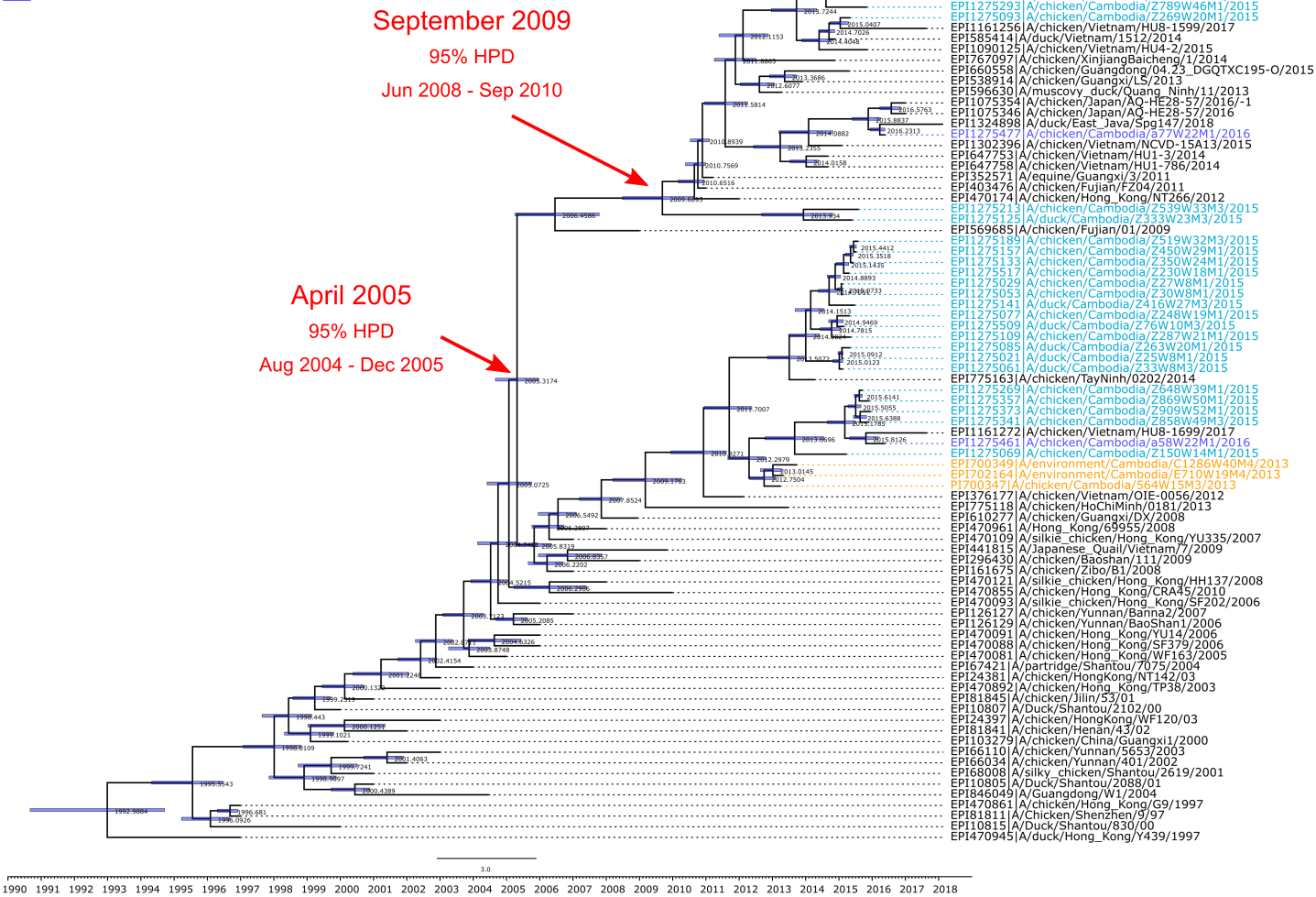

Supplement: S2 Fig — Cambodian AIVs are coloured based on the year of detection: 2013 is shown in orange, 2015 in green and 2016 in blue. Trees were produced using BEAST v1.84 with GTR + Γ and SRD06 nucleotide substitution model. The nodes are labelled with the proposed TMRCA and node bars show corresponding 95% HPD ranges. Branch lengths are time-proportional and the time scale is shown on the x-axis. (PDF) [file pone.0225428.s002.pdf]

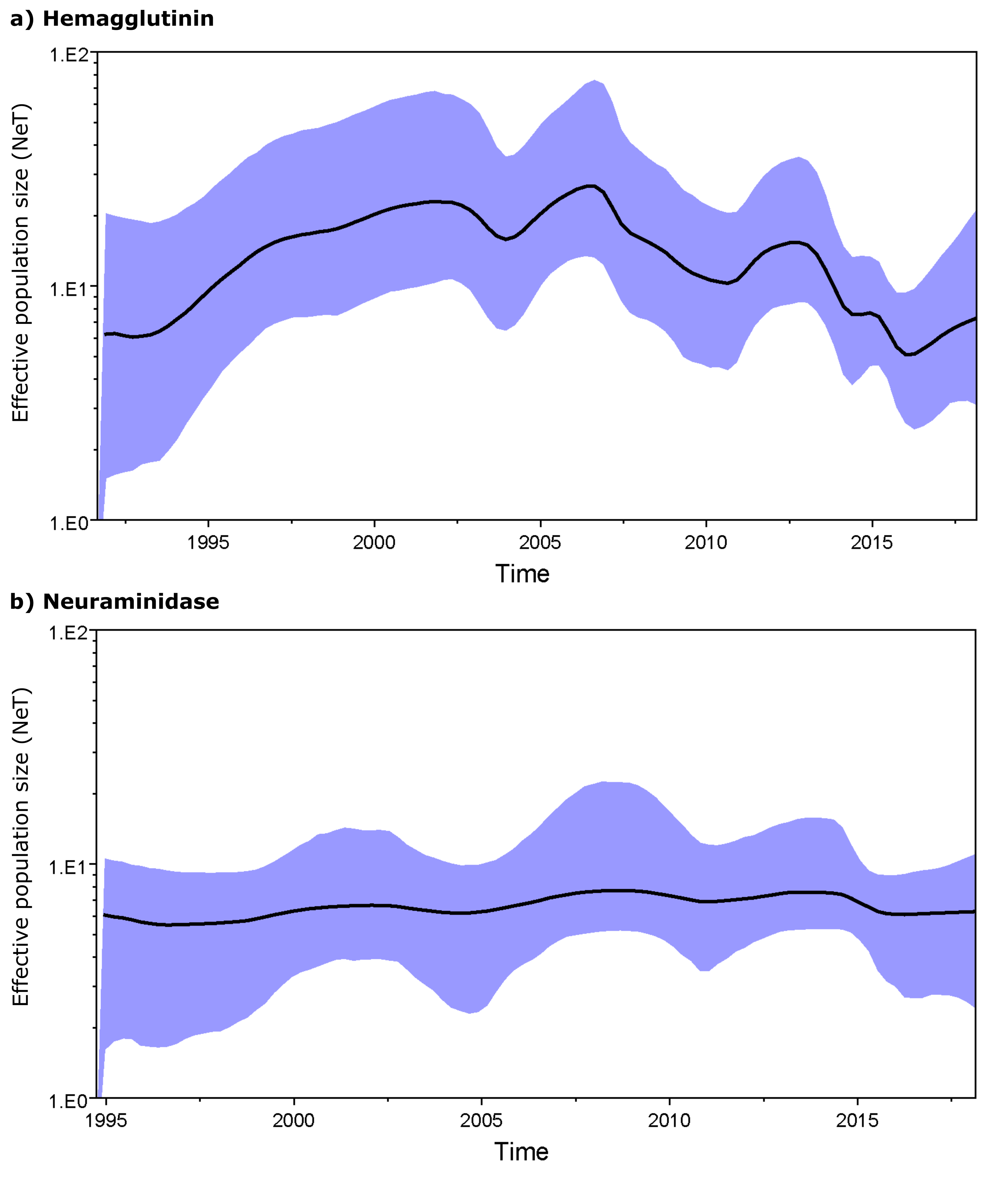

Supplement: S3 Fig — Genetic diversity of the HA and NA A(H9N2) genes was estimated using the Gaussian Markov Random Field (GMRF) model. The x-axis measures time in years and the y-axis is an estimate of genetic diversity calculated from Neτ (effective population size and the generation length in years) shown in log scale. The median estimate of genetic diversity over time is shown as a solid black line and the purple shading represents the 95% HPD intervals. (TIF) [file pone.0225428.s003.tif]
