# Supplementary material for: The evolution and genetic diversity of avian influenza A(H9N2) viruses in Cambodia, 2015 – 2016
Source: PLoS One. 2019 Dec 9;14(12):e0225428. doi: 10.1371/journal.pone.0225428 (PMC6901181; doi:10.1371/journal.pone.0225428)
Supplement: S4 Fig — a) PB2 b) PB1 c) PA d) NP e) MP and f) NS. Trees were generated with IQ-Tree using the GTR+ I + Γ model with 1,000 ultrafast boostrap replicates. Cambodian viruses are coloured based on the year of detection: 2015 is light blue and 2016 dark blue. AIVs identified in humans are coloured red, and G57 lineage viruses (as defined by Pu et al., 2015) are pink. Candidate vaccine viruses are indicated by an asterisks (*) next to the taxa name. Viruses from A(H9) reference lineages are shown in bold and the lineage Cambodian A(H9N2) viruses fall under is indicated on the right hand side of the tree. Bootstrap values of 70 or greater are displayed on branches. The scale bar indicates number of nucleotide substitutions per site. (PDF) [file pone.0225428.s004.pdf]

S4a) PB2

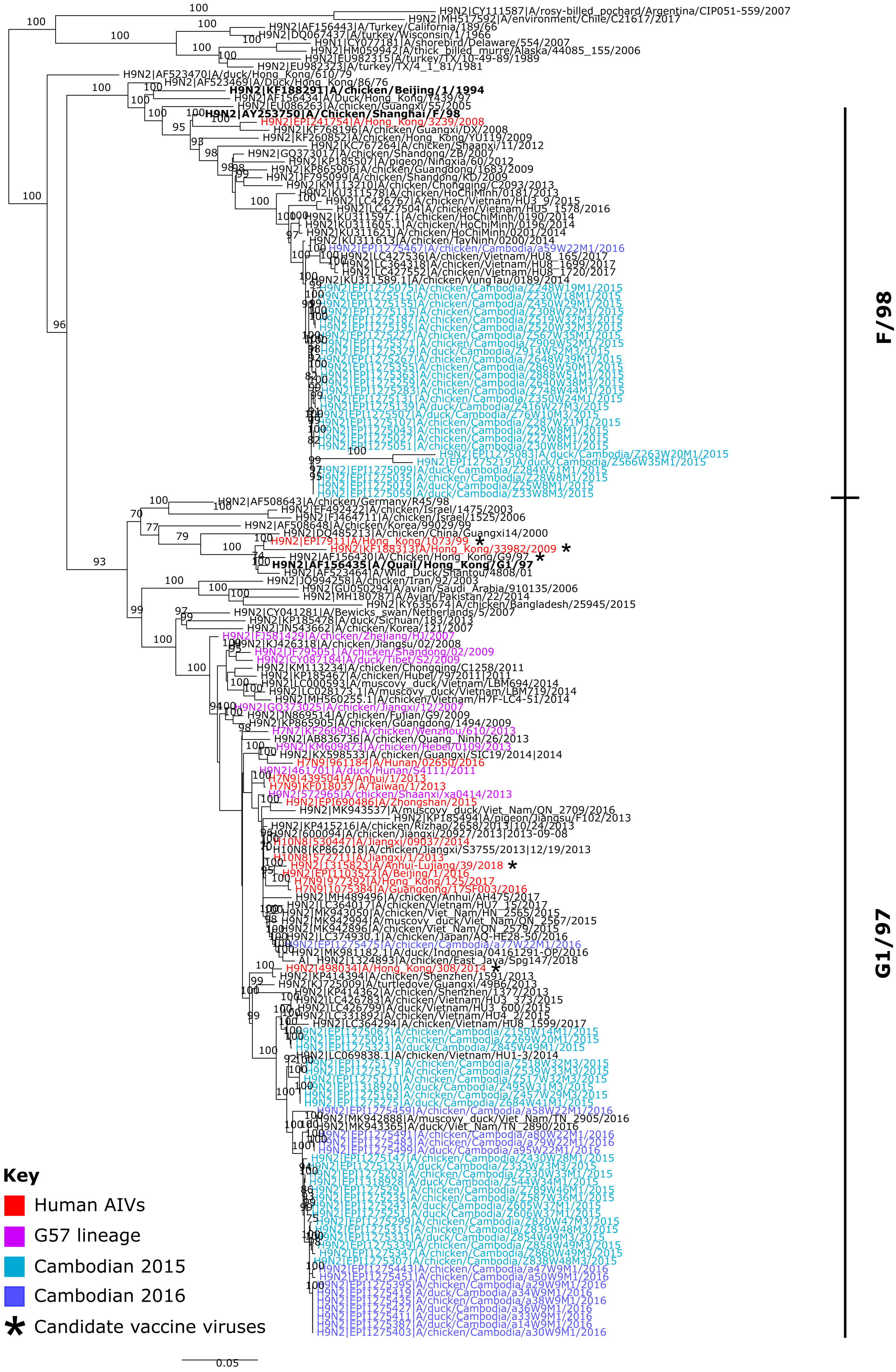
F/98
G1/97
0.05
Key
Human AIVs
G57 lineage
Cambodian 2015
Cambodian 2016
Candidate vaccine viruses

S4b) PB1

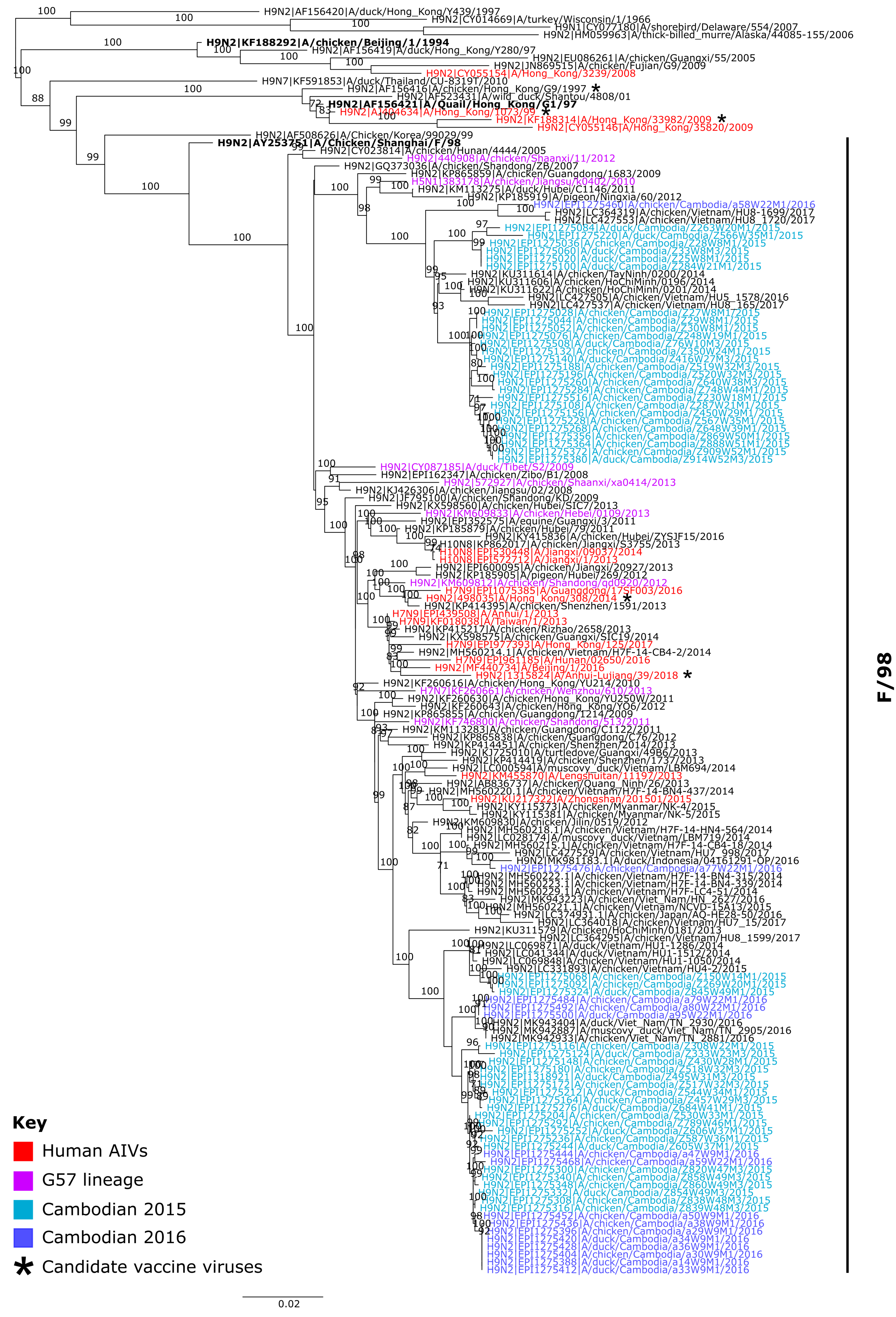
F/98
0.02
Key
Human AIVs
G57 lineage
Cambodian 2015
Cambodian 2016
Candidate vaccine viruses

S4c) PA

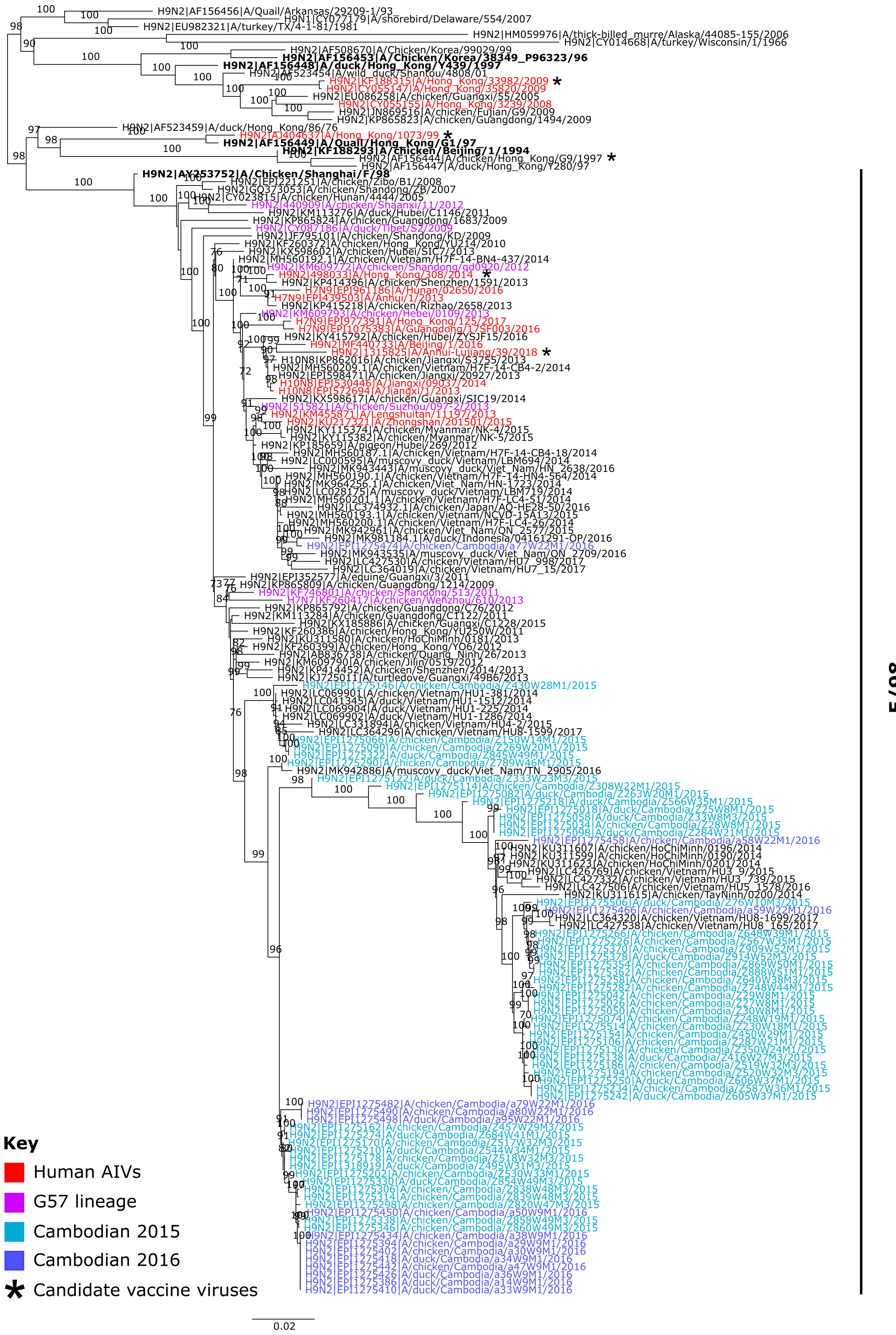
H9N2|AF156456|A/Quail/Arkansas/29209-1/93
H9N1|CY077179|A/shorebird/Delaware/554/2007
H9N2|EU982321|A/turkey/TX/4-1-81/1981
H9N2|HM059976|A/thick-billed_murre/Alaska/44085-155/2006
H9N2|CY014668|A/turkey/Wisconsin/1/1966
H9N2|AF508670|A/Chicken/Korea/99029/99
H9N2|AF156453|A/Chicken/Korea/38349_P96323/96
H9N2|AF156448|A/duck/Hong_Kong/Y439/1997
H9N2|AF523454|A/wild_duck/Shantou/4808/01
H9N2|KF188315|A/Hong_Kong/33982/2009 *
H9N2|CY055147|A/Hong_Kong/35820/2009
H9N2|EU086258|A/chicken/Guangxi/55/2005
H9N2|CY055155|A/Hong_Kong/3239/2008
H9N2|JN869516|A/chicken/Fujian/G9/2009
H9N2|KP865823|A/chicken/Guangdong/1494/2009
H9N2|AF523459|A/duck/Hong_Kong/86/76
H9N2|AJ404637|A/Hong_Kong/1073/99 *
H9N2|AF156449|A/Quail/Hong_Kong/G1/97
H9N2|KF188293|A/chicken/Beijing/1/1994
H9N2|AF156444|A/chicken/Hong_Kong/G9/1997 *
H9N2|AF156447|A/duck/Hong_Kong/Y280/97
H9N2|AY253752|A/Chicken/Shanghai/F/98
H9N2|EPI221251|A/chicken/Zibo/B1/2008
H9N2|GQ373053|A/chicken/Shandong/ZB/2007
H9N2|CY023815|A/chicken/Hunan/4444/2005
H9N2|440909|A/chicken/Shaanxi/11/2012
H9N2|KM113276|A/duck/Hubei/C1146/2011
H9N2|KP865824|A/chicken/Guangdong/1683/2009
H9N2|CY087186|A/duck/Tibet/S2/2009
H9N2|JF795101|A/chicken/Shandong/KD/2009
H9N2|KF260372|A/chicken/Hong_Kong/YU214/2010
H9N2|KX598602|A/chicken/Hubei/SIC7/2013
H9N2|MH560192.1|A/chicken/Vietnam/H7F-14-BN4-437/2014
H9N2|KM609772|A/chicken/Shandong/qd0920/2012
H9N2|498033|A/Hong_Kong/308/2014 *
H9N2|KP414396|A/chicken/Shenzhen/1591/2013
H7N9|EPI961186|A/Hunan/02650/2016
H7N9|EPI439503|A/Anhui/1/2013
H9N2|KP415218|A/chicken/Rizhao/2658/2013
H9N2|KM609793|A/chicken/Hebei/0109/2013
H7N9|EPI977391|A/Hong_Kong/125/2017
H7N9|EPI1075383|A/Guangdong/17SF003/2016
H9N2|KY415792|A/chicken/Hubei/ZYSJF15/2016
H9N2|MF440733|A/Beijing/1/2016
H9N2|1315825|A/Anhui-Lujiang/39/2018 *
H10N8|KP862016|A/chicken/Jiangxi/S3755/2013
H9N2|MH560209.1|A/chicken/Vietnam/H7F-14-CB4-2/2014
H9N2|EPI598471|A/chicken/Jiangxi/20927/2013
H10N8|EPI530446|A/Jiangxi/09037/2014
H10N8|EPI572694|A/Jiangxi/1/2013
H9N2|KX598617|A/chicken/Guangxi/SIC19/2014
H9N2|515821|A/Chicken/Suzhou/097-2/2013
H9N2|KM455871|A/Lengshuitan/11197/2013
H9N2|KU217321|A/Zhongshan/201501/2015
H9N2|KY115374|A/chicken/Myanmar/NK-4/2015
H9N2|KY115382|A/chicken/Myanmar/NK-5/2015
H9N2|KP185659|A/pigeon/Hubei/269/2012
H9N2|MH560187.1|A/chicken/Vietnam/H7F-14-CB4-18/2014
H9N2|LC000595|A/muscovy_duck/Vietnam/LBM694/2014
H9N2|MK943443|A/muscovy_duck/Viet_Nam/HN_2638/2016
H9N2|MH560190.1|A/chicken/Vietnam/H7F-14-HN4-564/2014
H9N2|MK964256.1|A/chicken/Viet_Nam/HN-1723/2014
H9N2|LC028175|A/muscovy_duck/Vietnam/LBM719/2014
H9N2|MH560201.1|A/chicken/Vietnam/H7F-LC4-51/2014
H9N2|LC374932.1|A/chicken/Japan/AQ-HE28-50/2016
H9N2|MH560193.1|A/chicken/Vietnam/NCVD-15A13/2015
H9N2|MH560200.1|A/chicken/Vietnam/H7F-LC4-26/2014
H9N2|MK942961|A/chicken/Viet_Nam/QN_2577/2015
H9N2|MK981184.1|A/duck/Indonesia/04161291-OP/2016
H9N2|EPI1275474|A/chicken/Cambodia/a77W22M1/2016
H9N2|MK943535|A/muscovy_duck/Viet_Nam/QN_2709/2016
H9N2|LC427530|A/chicken/Vietnam/HU7_998/2017
H9N2|LC364019|A/chicken/Vietnam/HU7_15/2017
H9N2|EPI352577|A/equine/Guangxi/3/2011
H9N2|KP865809|A/chicken/Guangdong/1214/2009
H9N2|KF746801|A/chicken/Shandong/513/2011
H7N7|KF260417|A/chicken/Wenzhou/610/2013
H9N2|KP865792|A/chicken/Guangdong/C76/2012
H9N2|KM113284|A/chicken/Guangdong/C1122/2011
H9N2|KX185886|A/chicken/Guangxi/C1228/2015
H9N2|KF260386|A/chicken/Hong_Kong/YU250W/2011
H9N2|KU311580|A/chicken/HoChiMinh/0181/2013
H9N2|KF260399|A/chicken/Hong_Kong/YO6/2012
H9N2|AB836738|A/chicken/Quang_Ninh/26/2013
H9N2|KM609790|A/chicken/Jilin/0519/2012
H9N2|KP414452|A/chicken/Shenzhen/2014/2013
H9N2|KJ725011|A/turtledove/Guangxi/49B6/2013
H9N2|EPI1275146|A/chicken/Cambodia/Z430W28M1/2015
H9N2|LC069901|A/chicken/Vietnam/HU1-381/2014
H9N2|LC041345|A/duck/Vietnam/HU1-1512/2014
H9N2|LC069904|A/duck/Vietnam/HU1-225/2014
H9N2|LC069902|A/duck/Vietnam/HU1-1286/2014
H9N2|LC331894|A/chicken/Vietnam/HU4-2/2015
H9N2|LC364296|A/chicken/Vietnam/HU8-1599/2017
H9N2|EPI1275066|A/chicken/Cambodia/Z150W14M1/2015
H9N2|EPI1275090|A/chicken/Cambodia/Z269W20M1/2015
H9N2|EPI1275322|A/duck/Cambodia/Z845W49M1/2015
H9N2|EPI1275290|A/chicken/Cambodia/Z789W46M1/2015
H9N2|MK942886|A/muscovy_duck/Viet_Nam/TN_2905/2016
H9N2|EPI1275122|A/duck/Cambodia/Z333W23M3/2015
H9N2|EPI1275114|A/chicken/Cambodia/Z308W22M1/2015
H9N2|EPI1275082|A/duck/Cambodia/Z263W20M1/2015
H9N2|EPI1275218|A/duck/Cambodia/Z566W35M1/2015
H9N2|EPI1275018|A/duck/Cambodia/Z25W8M1/2015
H9N2|EPI1275058|A/duck/Cambodia/Z33W8M3/2015
H9N2|EPI1275034|A/chicken/Cambodia/Z28W8M1/2015
H9N2|EPI1275098|A/duck/Cambodia/Z284W21M1/2015
H9N2|EPI1275458|A/chicken/Cambodia/a58W22M1/2016
H9N2|KU311607|A/chicken/HoChiMinh/0196/2014
H9N2|KU311599|A/chicken/HoChiMinh/0190/2014
H9N2|KU311623|A/chicken/HoChiMinh/0201/2014
H9N2|LC426769|A/chicken/Vietnam/HU3_9/2015
H9N2|LC427332|A/chicken/Vietnam/HU3_739/2015
H9N2|LC427506|A/chicken/Vietnam/HU5_1578/2016
H9N2|KU311615|A/chicken/TayNinh/0200/2014
H9N2|EPI1275506|A/duck/Cambodia/Z76W10M3/2015
H9N2|EPI1275466|A/duck/Cambodia/a59W22M1/2016
H9N2|LC364320|A/chicken/Vietnam/HU8-1699/2017
H9N2|LC427538|A/chicken/Vietnam/HU8_165/2017
H9N2|EPI1275266|A/chicken/Cambodia/Z648W39M1/2015
H9N2|EPI1275226|A/chicken/Cambodia/Z567W35M1/2015
H9N2|EPI1275370|A/chicken/Cambodia/Z909W52M1/2015
H9N2|EPI1275378|A/duck/Cambodia/Z914W52M3/2015
H9N2|EPI1275354|A/chicken/Cambodia/Z869W50M1/2015
H9N2|EPI1275362|A/chicken/Cambodia/Z888W51M1/2015
H9N2|EPI1275258|A/chicken/Cambodia/Z640W38M3/2015
H9N2|EPI1275282|A/chicken/Cambodia/Z748W44M1/2015
H9N2|EPI1275042|A/chicken/Cambodia/Z29W8M1/2015
H9N2|EPI1275026|A/chicken/Cambodia/Z27W8M1/2015
H9N2|EPI1275050|A/chicken/Cambodia/Z30W8M1/2015
H9N2|EPI1275074|A/chicken/Cambodia/Z248W19M1/2015
H9N2|EPI1275514|A/chicken/Cambodia/Z230W18M1/2015
H9N2|EPI1275154|A/chicken/Cambodia/Z450W29M1/2015
H9N2|EPI1275106|A/chicken/Cambodia/Z287W21M1/2015
H9N2|EPI1275130|A/chicken/Cambodia/Z350W24M1/2015
H9N2|EPI1275138|A/duck/Cambodia/Z416W27M3/2015
H9N2|EPI1275186|A/chicken/Cambodia/Z519W32M3/2015
H9N2|EPI1275194|A/chicken/Cambodia/Z520W32M3/2015
H9N2|EPI1275250|A/duck/Cambodia/Z606W37M1/2015
H9N2|EPI1275234|A/chicken/Cambodia/Z587W36M1/2015
H9N2|EPI1275242|A/duck/Cambodia/Z605W37M1/2015
H9N2|EPI1275482|A/chicken/Cambodia/a79W22M1/2016
H9N2|EPI1275490|A/chicken/Cambodia/a80W22M1/2016
H9N2|EPI1275498|A/duck/Cambodia/a95W22M1/2016
H9N2|EPI1275162|A/chicken/Cambodia/Z457W29M3/2015
H9N2|EPI1275274|A/duck/Cambodia/Z684W41M1/2015
H9N2|EPI1275170|A/chicken/Cambodia/Z517W32M3/2015
H9N2|EPI1275210|A/duck/Cambodia/Z544W34M1/2015
H9N2|EPI1275178|A/chicken/Cambodia/Z518W32M3/2015
H9N2|EPI1318919|A/duck/Cambodia/Z495W31M3/2015
H9N2|EPI1275202|A/chicken/Cambodia/Z530W33M1/2015
H9N2|EPI1275330|A/duck/Cambodia/Z854W49M3/2015
H9N2|EPI1275306|A/chicken/Cambodia/Z838W48M3/2015
H9N2|EPI1275314|A/chicken/Cambodia/Z839W48M3/2015
H9N2|EPI1275298|A/chicken/Cambodia/Z820W47M3/2015
H9N2|EPI1275450|A/chicken/Cambodia/a50W9M1/2016
H9N2|EPI1275338|A/chicken/Cambodia/Z858W49M3/2015
H9N2|EPI1275346|A/chicken/Cambodia/Z860W49M3/2015
H9N2|EPI1275434|A/chicken/Cambodia/a38W9M1/2016
H9N2|EPI1275394|A/chicken/Cambodia/a29W9M1/2016
H9N2|EPI1275402|A/chicken/Cambodia/a30W9M1/2016
H9N2|EPI1275418|A/duck/Cambodia/a34W9M1/2016
H9N2|EPI1275442|A/chicken/Cambodia/a47W9M1/2016
H9N2|EPI1275426|A/duck/Cambodia/a36W9M1/2016
H9N2|EPI1275386|A/duck/Cambodia/a14W9M1/2016
H9N2|EPI1275410|A/duck/Cambodia/a33W9M1/2016
F/98
0.02
Key
Human AIVs
G57 lineage
Cambodian 2015
Cambodian 2016
* Candidate vaccine viruses

S4d) NS

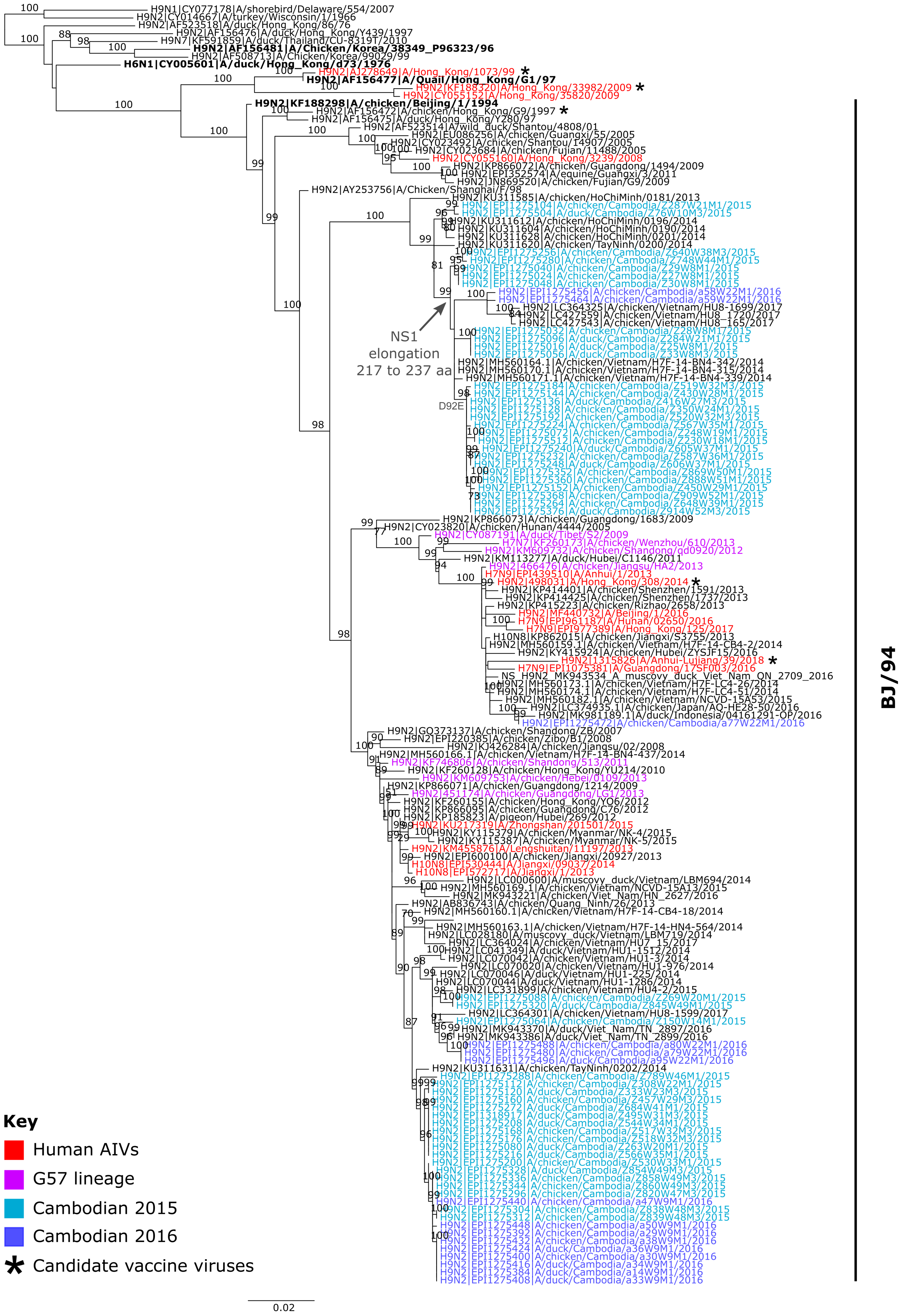
NS1
elongation
217 to 237 aa
D92E
BJ/94
0.02
Key
Human AIVs
G57 lineage
Cambodian 2015
Cambodian 2016
Candidate vaccine viruses

S4e) MP

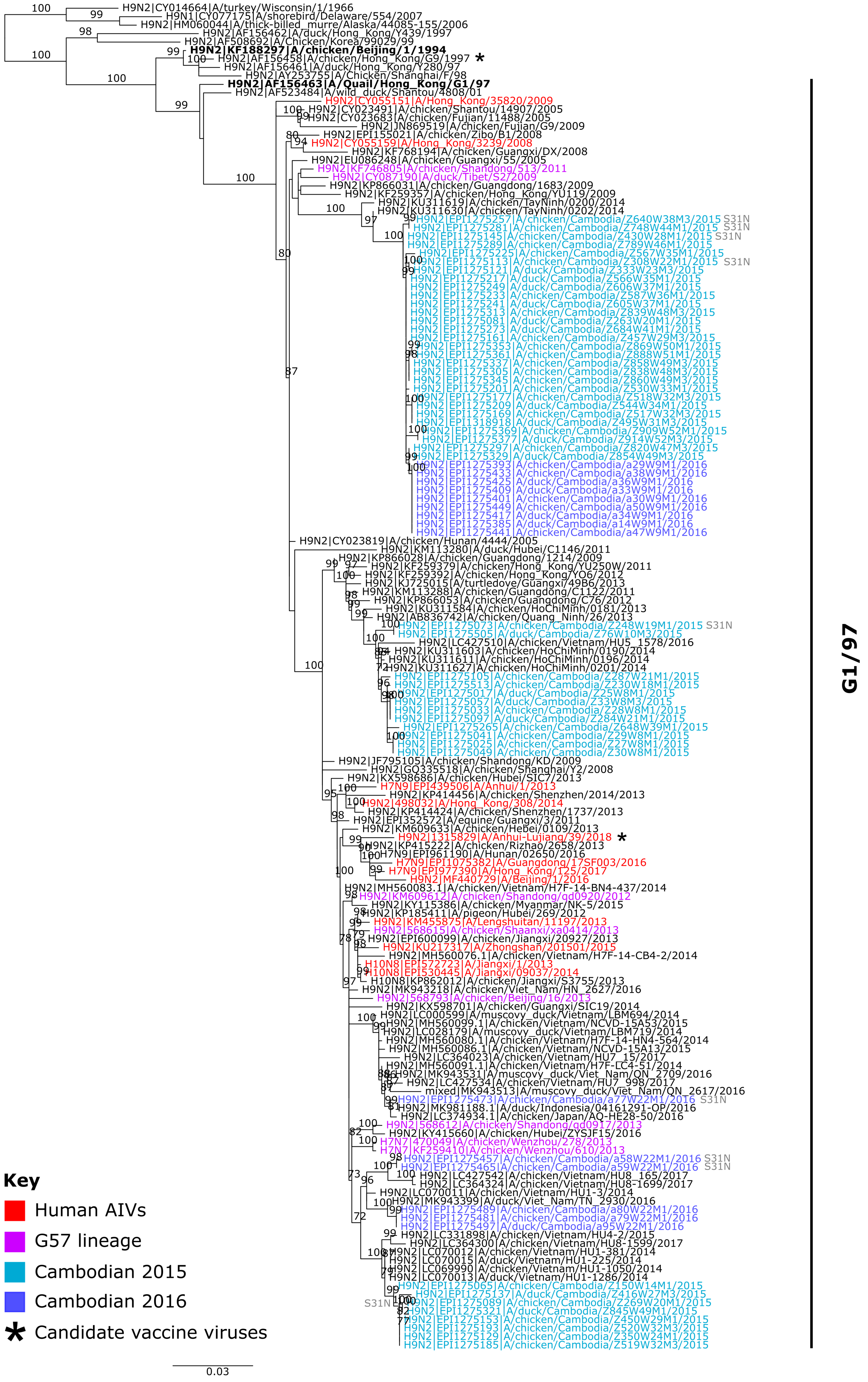

H9N2|CY014664|A/turkey/Wisconsin/1/1966
H9N1|CY077175|A/shorebird/Delaware/554/2007
H9N2|HM060044|A/thick-billed_murre/Alaska/44085-155/2006
H9N2|AF156462|A/duck/Hong_Kong/Y439/1997
H9N2|AF508692|A/Chicken/Korea/99029/99
H9N2|KF188297|A/chicken/Beijing/1/1994
H9N2|AF156458|A/chicken/Hong_Kong/G9/1997 *
H9N2|AF156461|A/duck/Hong_Kong/Y280/97
H9N2|AY253755|A/Chicken/Shanghai/F/98
H9N2|AF156463|A/Quail/Hong_Kong/G1/97
H9N2|AF523484|A/wild_duck/Shantou/4808/01
H9N2|CY055151|A/Hong_Kong/35820/2009
H9N2|CY023491|A/chicken/Shantou/14907/2005
H9N2|CY023683|A/chicken/Fujian/11488/2005
H9N2|JN869519|A/chicken/Fujian/G9/2009
H9N2|EPI155021|A/chicken/Zibo/B1/2008
H9N2|CY055159|A/Hong_Kong/3239/2008
H9N2|KF768194|A/chicken/Guangxi/DX/2008
H9N2|EU086248|A/chicken/Guangxi/55/2005
H9N2|KF746805|A/chicken/Shandong/513/2011
H9N2|CY087190|A/duck/Tibet/S2/2009
H9N2|KP866031|A/chicken/Guangdong/1683/2009
H9N2|KF259357|A/chicken/Hong_Kong/YU119/2009
H9N2|KU311619|A/chicken/TayNinh/0200/2014
H9N2|KU311630|A/chicken/TayNinh/0202/2014
H9N2|EPI1275257|A/chicken/Cambodia/Z640W38M3/2015 S31N
H9N2|EPI1275281|A/chicken/Cambodia/Z748W44M1/2015 S31N
H9N2|EPI1275145|A/chicken/Cambodia/Z430W28M1/2015 S31N
H9N2|EPI1275289|A/chicken/Cambodia/Z789W46M1/2015
H9N2|EPI1275225|A/chicken/Cambodia/Z567W35M1/2015
H9N2|EPI1275113|A/chicken/Cambodia/Z308W22M1/2015 S31N
H9N2|EPI1275121|A/duck/Cambodia/Z333W23M3/2015
H9N2|EPI1275217|A/duck/Cambodia/Z566W35M1/2015
H9N2|EPI1275249|A/duck/Cambodia/Z606W37M1/2015
H9N2|EPI1275233|A/chicken/Cambodia/Z587W36M1/2015
H9N2|EPI1275241|A/duck/Cambodia/Z605W37M1/2015
H9N2|EPI1275313|A/chicken/Cambodia/Z839W48M3/2015
H9N2|EPI1275081|A/duck/Cambodia/Z263W20M1/2015
H9N2|EPI1275273|A/duck/Cambodia/Z684W41M1/2015
H9N2|EPI1275161|A/chicken/Cambodia/Z457W29M3/2015
H9N2|EPI1275353|A/chicken/Cambodia/Z869W50M1/2015
H9N2|EPI1275361|A/chicken/Cambodia/Z888W51M1/2015
H9N2|EPI1275337|A/chicken/Cambodia/Z858W49M3/2015
H9N2|EPI1275305|A/chicken/Cambodia/Z838W48M3/2015
H9N2|EPI1275345|A/chicken/Cambodia/Z860W49M3/2015
H9N2|EPI1275201|A/chicken/Cambodia/Z530W33M1/2015
H9N2|EPI1275177|A/chicken/Cambodia/Z518W32M3/2015
H9N2|EPI1275209|A/duck/Cambodia/Z544W34M1/2015
H9N2|EPI1275169|A/chicken/Cambodia/Z517W32M3/2015
H9N2|EPI1318918|A/duck/Cambodia/Z495W31M3/2015
H9N2|EPI1275369|A/chicken/Cambodia/Z909W52M1/2015
H9N2|EPI1275377|A/duck/Cambodia/Z914W52M3/2015
H9N2|EPI1275297|A/chicken/Cambodia/Z820W47M3/2015
H9N2|EPI1275329|A/duck/Cambodia/Z854W49M3/2015
H9N2|EPI1275393|A/chicken/Cambodia/a29W9M1/2016
H9N2|EPI1275433|A/chicken/Cambodia/a38W9M1/2016
H9N2|EPI1275425|A/duck/Cambodia/a36W9M1/2016
H9N2|EPI1275409|A/duck/Cambodia/a33W9M1/2016
H9N2|EPI1275401|A/chicken/Cambodia/a30W9M1/2016
H9N2|EPI1275449|A/chicken/Cambodia/a50W9M1/2016
H9N2|EPI1275417|A/duck/Cambodia/a34W9M1/2016
H9N2|EPI1275385|A/duck/Cambodia/a14W9M1/2016
H9N2|EPI1275441|A/chicken/Cambodia/a47W9M1/2016
H9N2|CY023819|A/chicken/Hunan/4444/2005
H9N2|KM113280|A/duck/Hubei/C1146/2011
H9N2|KP866028|A/chicken/Guangdong/1214/2009
H9N2|KF259379|A/chicken/Hong_Kong/YU250W/2011
H9N2|KF259392|A/chicken/Hong_Kong/YO6/2012
H9N2|KJ725015|A/turtledove/Guangxi/49B6/2013
H9N2|KM113288|A/chicken/Guangdong/C1122/2011
H9N2|KP866053|A/chicken/Guangdong/C76/2012
H9N2|KU311584|A/chicken/HoChiMinh/0181/2013
H9N2|AB836742|A/chicken/Quang_Ninh/26/2013
H9N2|EPI1275073|A/chicken/Cambodia/Z248W19M1/2015 S31N
H9N2|EPI1275505|A/duck/Cambodia/Z76W10M3/2015
H9N2|LC427510|A/chicken/Vietnam/HU5_1578/2016
H9N2|KU311603|A/chicken/HoChiMinh/0190/2014
H9N2|KU311611|A/chicken/HoChiMinh/0196/2014
H9N2|KU311627|A/chicken/HoChiMinh/0201/2014
H9N2|EPI1275105|A/chicken/Cambodia/Z287W21M1/2015
H9N2|EPI1275513|A/chicken/Cambodia/Z230W18M1/2015
H9N2|EPI1275017|A/duck/Cambodia/Z25W8M1/2015
H9N2|EPI1275057|A/duck/Cambodia/Z33W8M3/2015
H9N2|EPI1275033|A/chicken/Cambodia/Z28W8M1/2015
H9N2|EPI1275097|A/duck/Cambodia/Z284W21M1/2015
H9N2|EPI1275265|A/chicken/Cambodia/Z648W39M1/2015
H9N2|EPI1275041|A/chicken/Cambodia/Z29W8M1/2015
H9N2|EPI1275025|A/chicken/Cambodia/Z27W8M1/2015
H9N2|EPI1275049|A/chicken/Cambodia/Z30W8M1/2015
H9N2|JF795105|A/chicken/Shandong/KD/2009
H9N2|GQ335518|A/chicken/Shanghai/Y2/2008
H9N2|KX598686|A/chicken/Hubei/SIC7/2013
H7N9|EPI439506|A/Anhui/1/2013
H9N2|KP414456|A/chicken/Shenzhen/2014/2013
H9N2|498032|A/Hong_Kong/308/2014
H9N2|KP414424|A/chicken/Shenzhen/1737/2013
H9N2|EPI352572|A/equine/Guangxi/3/2011
H9N2|KM609633|A/chicken/Hebei/0109/2013
H9N2|1315829|A/Anhui-Lujiang/39/2018 *
H9N2|KP415222|A/chicken/Rizhao/2658/2013
H7N9|EPI961190|A/Hunan/02650/2016
H7N9|EPI1075382|A/Guangdong/17SF003/2016
H7N9|EPI977390|A/Hong_Kong/125/2017
H9N2|MF440729|A/Beijing/1/2016
H9N2|MH560083.1|A/chicken/Vietnam/H7F-14-BN4-437/2014
H9N2|KM609612|A/chicken/Shandong/gd0920/2012
H9N2|KY115386|A/chicken/Myanmar/NK-5/2015
H9N2|KP185411|A/pigeon/Hubei/269/2012
H9N2|KM455875|A/Lengshuitan/11197/2013
H9N2|568615|A/chicken/Shaanxi/xa0414/2013
H9N2|EPI600099|A/chicken/Jiangxi/20927/2013
H9N2|KU217317|A/Zhongshan/201501/2015
H9N2|MH560076.1|A/chicken/Vietnam/H7F-14-CB4-2/2014
H10N8|EPI572723|A/Jiangxi/1/2013
H10N8|EPI530445|A/Jiangxi/09037/2014
H10N8|KP862012|A/chicken/Jiangxi/S3755/2013
H9N2|MK943218|A/chicken/Viet_Nam/HN_2627/2016
H9N2|568793|A/chicken/Beijing/16/2013
H9N2|KX598701|A/chicken/Guangxi/SIC19/2014
H9N2|LC000599|A/muscovy_duck/Vietnam/LBM694/2014
H9N2|MH560099.1|A/chicken/Vietnam/NCVD-15A53/2015
H9N2|LC028179|A/muscovy_duck/Vietnam/LBM719/2014
H9N2|MH560080.1|A/chicken/Vietnam/H7F-14-HN4-564/2014
H9N2|MH560086.1|A/chicken/Vietnam/NCVD-15A13/2015
H9N2|LC364023|A/chicken/Vietnam/HU7_15/2017
H9N2|MH560091.1|A/chicken/Vietnam/H7F-LC4-51/2014
H9N2|MK943531|A/muscovy_duck/Viet_Nam/QN_2709/2016
H9N2|LC427534|A/chicken/Vietnam/HU7_998/2017
mixed|MK943513|A/muscovy_duck/Viet_Nam/QN_2617/2016
H9N2|EPI1275473|A/chicken/Cambodia/a77W22M1/2016 S31N
H9N2|MK981188.1|A/duck/Indonesia/04161291-OP/2016
H9N2|LC374934.1|A/chicken/Japan/AQ-HE28-50/2016
H9N2|568612|A/chicken/Shandong/gd0917/2013
H9N2|KY415660|A/chicken/Hubei/ZYSJF15/2016
H7N7|470049|A/chicken/Wenzhou/278/2013
H7N7|KF259410|A/chicken/Wenzhou/610/2013
H9N2|EPI1275457|A/chicken/Cambodia/a58W22M1/2016 S31N
H9N2|EPI1275465|A/chicken/Cambodia/a59W22M1/2016 S31N
H9N2|LC427542|A/chicken/Vietnam/HU8_165/2017
H9N2|LC364324|A/chicken/Vietnam/HU8-1699/2017
H9N2|LC070011|A/chicken/Vietnam/HU1-3/2014
H9N2|MK943399|A/duck/Viet_Nam/TN_2930/2016
H9N2|EPI1275489|A/chicken/Cambodia/a80W22M1/2016
H9N2|EPI1275481|A/chicken/Cambodia/a79W22M1/2016
H9N2|EPI1275497|A/duck/Cambodia/a95W22M1/2016
H9N2|LC331898|A/chicken/Vietnam/HU4-2/2015
H9N2|LC364300|A/chicken/Vietnam/HU8-1599/2017
H9N2|LC070012|A/chicken/Vietnam/HU1-381/2014
H9N2|LC070015|A/duck/Vietnam/HU1-225/2014
H9N2|LC069990|A/chicken/Vietnam/HU1-1050/2014
H9N2|LC070013|A/duck/Vietnam/HU1-1286/2014
H9N2|EPI1275065|A/chicken/Cambodia/Z150W14M1/2015
H9N2|EPI1275137|A/duck/Cambodia/Z416W27M3/2015
S31N H9N2|EPI1275089|A/chicken/Cambodia/Z269W20M1/2015
H9N2|EPI1275321|A/duck/Cambodia/Z845W49M1/2015
H9N2|EPI1275153|A/chicken/Cambodia/Z450W29M1/2015
H9N2|EPI1275193|A/chicken/Cambodia/Z520W32M3/2015
H9N2|EPI1275129|A/chicken/Cambodia/Z350W24M1/2015
H9N2|EPI1275185|A/chicken/Cambodia/Z519W32M3/2015
G1/97
Key
Human AIVs
G57 lineage
Cambodian 2015
Cambodian 2016
Candidate vaccine viruses
0.03

S4f) NP

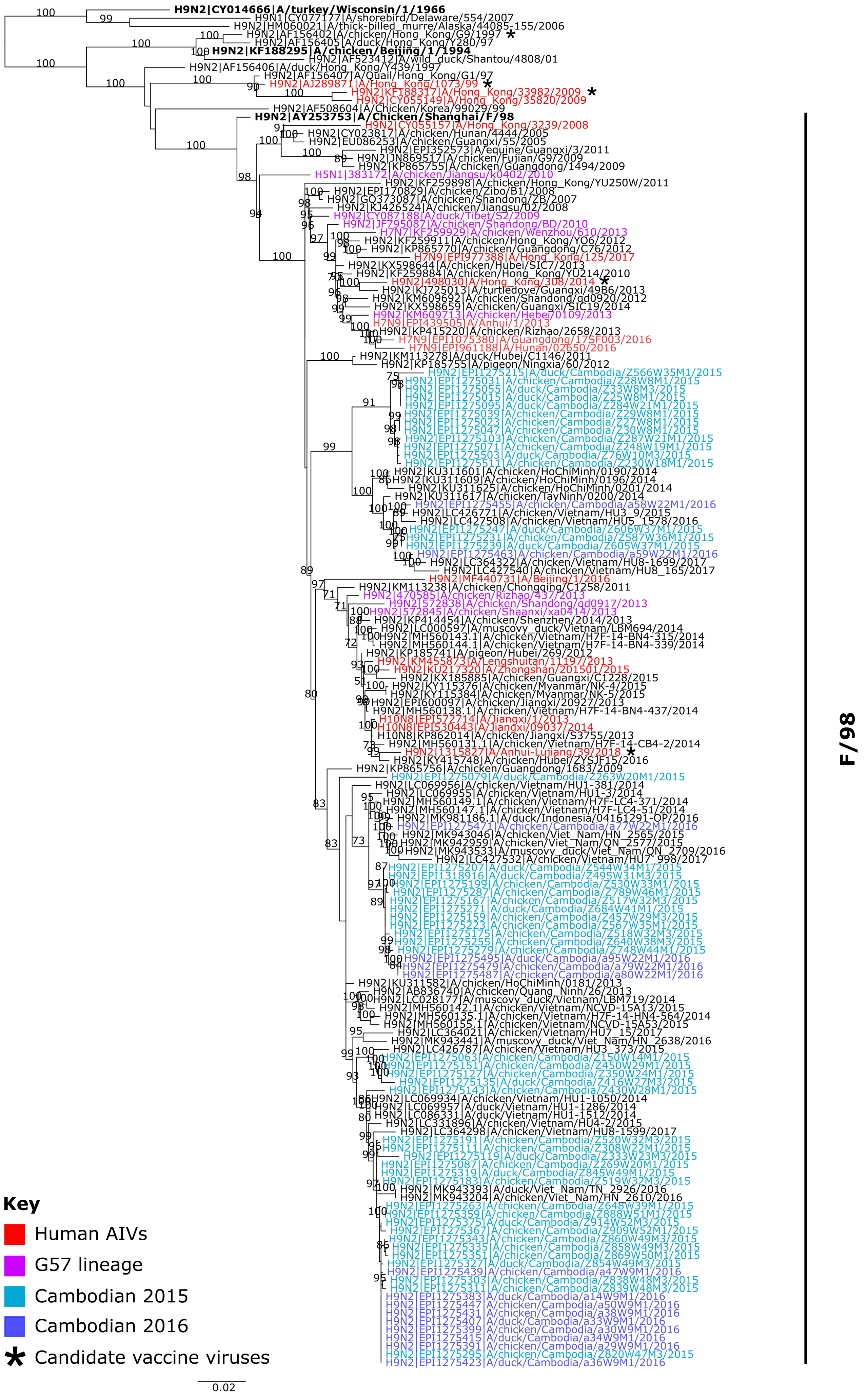
H9N2|CY014666|A/turkey/Wisconsin/1/1966
H9N1|CY077177|A/shorebird/Delaware/554/2007
H9N2|HM060021|A/thick-billed_murre/Alaska/44085-155/2006
H9N2|AF156402|A/chicken/Hong_Kong/G9/1997
H9N2|AF156405|A/duck/Hong_Kong/Y280/97
H9N2|KF188295|A/chicken/Beijing/1/1994
H9N2|AF523412|A/wild_duck/Shantou/4808/01
H9N2|AF156406|A/duck/Hong_Kong/Y439/1997
H9N2|AF156407|A/Quail/Hong_Kong/G1/97
H9N2|AJ289871|A/Hong_Kong/1073/99
H9N2|KF188317|A/Hong_Kong/33982/2009
H9N2|CY055149|A/Hong_Kong/35820/2009
H9N2|AF508604|A/Chicken/Korea/99029/99
H9N2|AY253753|A/Chicken/Shanghai/F/98
H9N2|CY055157|A/Hong_Kong/3239/2008
H9N2|CY023817|A/chicken/Hunan/4444/2005
H9N2|EU086253|A/chicken/Guangxi/55/2005
H9N2|EPI352573|A/equine/Guangxi/3/2011
H9N2|JN869517|A/chicken/Fujian/G9/2009
H9N2|KP865755|A/chicken/Guangdong/1494/2009
H5N1|383172|A/chicken/Jiangsu/k0402/2010
H9N2|KF259898|A/chicken/Hong_Kong/YU250W/2011
H9N2|EPI170829|A/chicken/Zibo/B1/2008
H9N2|GQ373087|A/chicken/Shandong/ZB/2007
H9N2|KJ426524|A/chicken/Jiangsu/02/2008
H9N2|CY087188|A/duck/Tibet/S2/2009
H9N2|JF795087|A/chicken/Shandong/BD/2010
H7N7|KF259929|A/chicken/Wenzhou/610/2013
H9N2|KF259911|A/chicken/Hong_Kong/YQ6/2012
H9N2|KP865770|A/chicken/Guangdong/C76/2012
H7N9|EPI977388|A/Hong_Kong/125/2017
H9N2|KX598644|A/chicken/Hubei/SIC7/2013
H9N2|KF259884|A/chicken/Hong_Kong/YU214/2010
H9N2|498030|A/Hong_Kong/308/2014
H9N2|KJ725013|A/turtledove/Guangxi/49B6/2013
H9N2|KM609692|A/chicken/Shandong/qd0920/2012
H9N2|KX598659|A/chicken/Guangxi/SIC19/2014
H9N2|KM609713|A/chicken/Hebei/0109/2013
H7N9|EPI439505|A/Anhui/1/2013
H9N2|KP415220|A/chicken/Rizhao/2658/2013
H7N9|EPI1075380|A/Guangdong/17SF003/2016
H7N9|EPI961188|A/Hunan/02650/2016
H9N2|KM113278|A/duck/Hubei/C1146/2011
H9N2|KP185755|A/pigeon/Ningxia/60/2012
H9N2|EPI1275215|A/duck/Cambodia/Z566W35M1/2015
H9N2|EPI1275031|A/chicken/Cambodia/Z28W8M1/2015
H9N2|EPI1275055|A/duck/Cambodia/Z33W8M3/2015
H9N2|EPI1275015|A/duck/Cambodia/Z25W8M1/2015
H9N2|EPI1275095|A/duck/Cambodia/Z284W21M1/2015
H9N2|EPI1275039|A/chicken/Cambodia/Z29W8M1/2015
H9N2|EPI1275023|A/chicken/Cambodia/Z27W8M1/2015
H9N2|EPI1275047|A/chicken/Cambodia/Z30W8M1/2015
H9N2|EPI1275103|A/chicken/Cambodia/Z287W21M1/2015
H9N2|EPI1275071|A/chicken/Cambodia/Z248W19M1/2015
H9N2|EPI1275503|A/duck/Cambodia/Z76W10M3/2015
H9N2|EPI1275511|A/chicken/Cambodia/Z230W18M1/2015
H9N2|KU311601|A/chicken/HoChiMinh/0190/2014
H9N2|KU311609|A/chicken/HoChiMinh/0196/2014
H9N2|KU311625|A/chicken/HoChiMinh/0201/2014
H9N2|KU311617|A/chicken/TayNinh/0200/2014
H9N2|EPI1275455|A/chicken/Cambodia/a58W22M1/2016
H9N2|LC426771|A/chicken/Vietnam/HU3_9/2015
H9N2|LC427508|A/chicken/Vietnam/HU5_1578/2016
H9N2|EPI1275247|A/duck/Cambodia/Z606W37M1/2015
H9N2|EPI1275231|A/chicken/Cambodia/Z587W36M1/2015
H9N2|EPI1275239|A/duck/Cambodia/Z605W37M1/2015
H9N2|EPI1275463|A/chicken/Cambodia/a59W22M1/2016
H9N2|LC364322|A/chicken/Vietnam/HU8-1699/2017
H9N2|LC427540|A/chicken/Vietnam/HU8_165/2017
H9N2|MF440731|A/Beijing/1/2016
H9N2|KM113238|A/chicken/Chongqing/C1258/2011
H9N2|470585|A/chicken/Rizhao/437/2013
H9N2|572838|A/chicken/Shandong/qd0917/2013
H9N2|572845|A/chicken/Shaanxi/xa0414/2013
H9N2|KP414454|A/chicken/Shenzhen/2014/2013
H9N2|LC000597|A/muscovy_duck/Vietnam/LBM694/2014
H9N2|MH560143.1|A/chicken/Vietnam/H7F-14-BN4-315/2014
H9N2|MH560144.1|A/chicken/Vietnam/H7F-14-BN4-339/2014
H9N2|KP185741|A/pigeon/Hubei/269/2012
H9N2|KM455873|A/Lengshuitan/11197/2013
H9N2|KU217320|A/Zhongshan/201501/2015
H9N2|KX185885|A/chicken/Guangxi/C1228/2015
H9N2|KY115376|A/chicken/Myanmar/NK-4/2015
H9N2|KY115384|A/chicken/Myanmar/NK-5/2015
H9N2|EPI600097|A/chicken/Jiangxi/20927/2013
H9N2|MH560138.1|A/chicken/Vietnam/H7F-14-BN4-437/2014
H10N8|EPI572714|A/Jiangxi/1/2013
H10N8|EPI530443|A/Jiangxi/09037/2014
H10N8|KP862014|A/chicken/Jiangxi/S3755/2013
H9N2|MH560131.1|A/chicken/Vietnam/H7F-14-CB4-2/2014
H9N2|1315827|A/Anhui-Lujiang/39/2018
H9N2|KY415748|A/chicken/Hubei/ZYSJF15/2016
H9N2|KP865756|A/chicken/Guangdong/1683/2009
H9N2|EPI1275079|A/duck/Cambodia/Z263W20M1/2015
H9N2|LC069956|A/chicken/Vietnam/HU1-381/2014
H9N2|LC069955|A/chicken/Vietnam/HU1-3/2014
H9N2|MH560149.1|A/chicken/Vietnam/H7F-LC4-371/2014
H9N2|MH560147.1|A/chicken/Vietnam/H7F-LC4-51/2014
H9N2|MK981186.1|A/duck/Indonesia/04161291-OP/2016
H9N2|EPI1275471|A/chicken/Cambodia/a77W22M1/2016
H9N2|MK943046|A/chicken/Viet_Nam/HN_2565/2015
H9N2|MK942959|A/chicken/Viet_Nam/QN_2577/2015
H9N2|MK943533|A/muscovy_duck/Viet_Nam/QN_2709/2016
H9N2|LC427532|A/chicken/Vietnam/HU7_998/2017
H9N2|EPI1275207|A/duck/Cambodia/Z544W34M1/2015
H9N2|EPI1318916|A/duck/Cambodia/Z495W31M3/2015
H9N2|EPI1275199|A/chicken/Cambodia/Z530W33M1/2015
H9N2|EPI1275287|A/chicken/Cambodia/Z789W46M1/2015
H9N2|EPI1275167|A/chicken/Cambodia/Z517W32M3/2015
H9N2|EPI1275271|A/duck/Cambodia/Z684W41M1/2015
H9N2|EPI1275159|A/chicken/Cambodia/Z457W29M3/2015
H9N2|EPI1275223|A/chicken/Cambodia/Z567W35M1/2015
H9N2|EPI1275175|A/chicken/Cambodia/Z518W32M3/2015
H9N2|EPI1275255|A/chicken/Cambodia/Z640W38M3/2015
H9N2|EPI1275279|A/chicken/Cambodia/Z748W44M1/2015
H9N2|EPI1275495|A/duck/Cambodia/a95W22M1/2016
H9N2|EPI1275479|A/chicken/Cambodia/a79W22M1/2016
H9N2|EPI1275487|A/chicken/Cambodia/a80W22M1/2016
H9N2|KU311582|A/chicken/HoChiMinh/0181/2013
H9N2|AB836740|A/chicken/Quang_Ninh/26/2013
H9N2|LC028177|A/muscovy_duck/Vietnam/LBM719/2014
H9N2|MH560142.1|A/chicken/Vietnam/NCVD-15A13/2015
H9N2|MH560135.1|A/chicken/Vietnam/H7F-14-HN4-564/2014
H9N2|MH560155.1|A/chicken/Vietnam/NCVD-15A53/2015
H9N2|LC364021|A/chicken/Vietnam/HU7_15/2017
H9N2|MK943441|A/muscovy_duck/Viet_Nam/HN_2638/2016
H9N2|LC426787|A/chicken/Vietnam/HU3_373/2015
H9N2|EPI1275063|A/chicken/Cambodia/Z150W14M1/2015
H9N2|EPI1275151|A/chicken/Cambodia/Z450W29M1/2015
H9N2|EPI1275127|A/chicken/Cambodia/Z350W24M1/2015
H9N2|EPI1275135|A/duck/Cambodia/Z416W27M3/2015
H9N2|EPI1275143|A/chicken/Cambodia/Z430W28M1/2015
H9N2|LC069934|A/chicken/Vietnam/HU1-1050/2014
H9N2|LC069957|A/duck/Vietnam/HU1-1286/2014
H9N2|LC086331|A/duck/Vietnam/HU1-1512/2014
H9N2|LC331896|A/chicken/Vietnam/HU4-2/2015
H9N2|LC364298|A/chicken/Vietnam/HU8-1599/2017
H9N2|EPI1275191|A/chicken/Cambodia/Z520W32M3/2015
H9N2|EPI1275111|A/chicken/Cambodia/Z308W22M1/2015
H9N2|EPI1275119|A/duck/Cambodia/Z333W23M3/2015
H9N2|EPI1275087|A/chicken/Cambodia/Z269W20M1/2015
H9N2|EPI1275319|A/duck/Cambodia/Z845W49M1/2015
H9N2|EPI1275183|A/chicken/Cambodia/Z519W32M3/2015
H9N2|MK943393|A/duck/Viet_Nam/TN_2926/2016
H9N2|MK943204|A/chicken/Viet_Nam/HN_2610/2016
H9N2|EPI1275263|A/chicken/Cambodia/Z648W39M1/2015
H9N2|EPI1275359|A/chicken/Cambodia/Z888W51M1/2015
H9N2|EPI1275375|A/duck/Cambodia/Z914W52M3/2015
H9N2|EPI1275367|A/chicken/Cambodia/Z909W52M1/2015
H9N2|EPI1275343|A/chicken/Cambodia/Z860W49M3/2015
H9N2|EPI1275335|A/chicken/Cambodia/Z858W49M3/2015
H9N2|EPI1275351|A/chicken/Cambodia/Z869W50M1/2015
H9N2|EPI1275327|A/duck/Cambodia/Z854W49M3/2015
H9N2|EPI1275439|A/chicken/Cambodia/a47W9M1/2016
H9N2|EPI1275303|A/chicken/Cambodia/Z838W48M3/2015
H9N2|EPI1275311|A/chicken/Cambodia/Z839W48M3/2015
H9N2|EPI1275383|A/duck/Cambodia/a14W9M1/2016
H9N2|EPI1275447|A/chicken/Cambodia/a50W9M1/2016
H9N2|EPI1275431|A/chicken/Cambodia/a38W9M1/2016
H9N2|EPI1275407|A/duck/Cambodia/a33W9M1/2016
H9N2|EPI1275399|A/chicken/Cambodia/a30W9M1/2016
H9N2|EPI1275415|A/duck/Cambodia/a34W9M1/2016
H9N2|EPI1275391|A/chicken/Cambodia/a29W9M1/2016
H9N2|EPI1275295|A/chicken/Cambodia/Z820W47M3/2015
H9N2|EPI1275423|A/duck/Cambodia/a36W9M1/2016
F/98
Key
Human AIVs
G57 lineage
Cambodian 2015
Cambodian 2016
Candidate vaccine viruses
0.02
